# Dissecting the propensity of RIM1 subdomains to form phase condensates

**DOI:** 10.1038/s41598-026-56567-6

**Published:** 2026-06-23

**Authors:** Luis Kersten, Maksim Galkov, Paulina Nemcova, Aya Hanaey Dandrawy Mostafa, Anne Quatraccioni, Dirk Dietrich, Susanne Schoch

**Affiliations:** Institute of Cellular Neurosciences II, University Hospital Bonn, University of Bonn, Venusberg Campus 1, 53127 Bonn, Germany

**Keywords:** Biochemistry, Biophysics, Cell biology, Structural biology

## Abstract

Emerging evidence indicates that liquid-liquid phase separation (LLPS) may orchestrate synaptic vesicle and active zone (AZ) protein organization in presynaptic terminals via biomolecular condensates. A protein’s LLPS propensity is determined by intrinsic factors such as structural disorder, amino acid composition, multivalent interactions, and regulation by post-translational modifications, highlighting the need for a systematic dissection of condensate-forming regions in a cellular context. Here, we systematically examined the condensation properties of the AZ protein RIM1 using an established cell-based overexpression assay and found that, while full-length RIM1 exhibits the highest condensation propensity in our assay, even fragments as short as ~ 250 amino acids form condensate-like assemblies in HEK cells. For all but four fragments, their propensity to be enriched in droplets over the intracellular solution quantitatively correlates with their length. We identified a minimal region that closely reproduces key full-length RIM1 droplet properties with regard to abundance per cell and volume, comprising the zinc finger domain, the intrinsically disordered region 1 (IDR1), the proline-rich motif 1 (PRM1), the PDZ- and C2A-domains, and two non-condensing regions, the sequences containing either PRM2 and IDR2 or PRM1 till the end of the C2A-domain. In contrast to in vitro data, RIM-BP2 promotes phase condensation of full-length RIM1 only at lower RIM1 expression levels and differentially impacts the phase condensation capacity of different RIM1 truncation mutants. Taken together, our results define the phase condensation behavior of different RIM1 sequence elements and show that enrichment of the protein in the phase condensates increases exponentially with the length of the fragments.

**Supplementary Information:**

The online version contains supplementary material available at 10.1038/s41598-026-56567-6.

## Introduction

Synapses in the brain are the key unit for information transmission and processing. Synaptic strength varies significantly between neurons and synapse types and depends on the number of fusion-ready synaptic vesicles (SVs) docked at a specialized region of the presynaptic plasma membrane, the active zone (AZ)^1^. Based on nanoscopic analyses, such as electron microscopy and super-resolution microscopy, it has been suggested that proteins at the AZ accumulate in discrete clusters^1–3^ and that there might be defined release sites within the AZ^4^. Six evolutionary conserved protein families that are highly enriched at the AZ and strongly interconnected by direct interactions are thought to be the key components of both release sites and protein nanoassemblies, and are important regulators of SV fusion^2,5–7^. However, the precise organization and composition of these subcellular supramolecular complexes, as well as the molecular mechanisms regulating their formation, remain to be elucidated.

Liquid-liquid phase separation (LLPS) is a process by which proteins undergo a phase transition to form hydrogels that constitute membraneless sub-compartments, while maintaining a dynamic equilibrium with the environment^8–10^. Recent evidence has brought forward the concept that the formation of membraneless compartments by LLPS is involved in the sub-compartmentalization of proteins at the AZ as one mechanism underlying the accumulation of molecules and the segregation of proteins within these condensates^2,3,11–14^. This idea is supported by the finding that in vitro, LLPS of purified AZ proteins, alone or in combination, was observed for all proteins tested so far: RIM1, RIM-BP, Liprin-α, Piccolo, and ELKS^15–18^. Furthermore, after transfection of non-neuronal cells, including HEK293T cells, with plasmids encoding the above-mentioned AZ proteins, cytoplasmic condensates/droplets with liquid-like properties are observed^16,19–21^. In the native disordered state, proteins are largely free to diffuse in the cellular environment, whereas in the droplet state, movement is restricted. It has been proposed that in neurons, AZ proteins will exist in both native and condensed states and will most likely have different functions depending on which state they are in. The formation of phase condensates depends on protein concentration and sequence composition and thus on the cellular environment, such as interactions with other proteins and post-translational modifications that alter the electrostatic properties of the protein^22–24^. This raises the question of how the phase transitions of AZ proteins are controlled. To answer this question, a better understanding of which protein regions might be involved in the LLPS of individual proteins and how they interact to control the properties of the full-length proteins is required.

RIM1 is a core AZ scaffolding protein that regulates synaptic vesicle release by tethering voltage-gated calcium channels (VGCCs) to AZs^25,26^, by docking and priming vesicles for release via its interaction with Munc13^27–29^, and by interacting with multiple presynaptic proteins such as the synaptic vesicle protein Rab3 and most other AZ proteins^1,7^. RIM1 is required for the induction of presynaptic long-term potentiation^30^ and homeostatic synaptic plasticity^31^, and contributes to forms of short-term plasticity^27^. Recent studies have revealed that RIM1 possesses intrinsic phase separation properties and, together with RIM-BP, Piccolo C-terminal region and ELKS, forms dynamic phase condensates that can recruit VGCCs and tether SVs on condensate surfaces^17,32^. It is currently still unresolved how the individual structural domains and intrinsically disordered regions (IDRs) of RIM1 contribute to the biophysical properties of RIM1 condensates, like the formation, size, and concentration of the protein in the droplets in live cells.

Biophysical analyses suggest that a protein’s propensity for phase separation is influenced by IDRs, amino acid regions that lack a well-defined structure^33–35^. In addition, protein segments with a less complex amino acid composition, known as low-complexity regions, typically facilitate weak protein interactions associated with liquid-liquid phase separation^36^. However, oligomerization via specific protein domains like coiled-coil can also facilitate phase condensation, as shown for oligomerized Liprin-α and ELKS^16^. In vivo, LLPS is controlled by several external factors, such as post-translational modifications, in particular phosphorylation^37,38^. Despite the development of several tools for predicting phase-separating proteins and the responsible domains based on the increasing available experimental data, the correlation between the predictions and the measured data still needs to be improved^39–41^. Therefore, it is necessary to determine the regions and domains that mediate LLPS in vivo for each individual protein.

The aim of the present study was to systematically dissect how different sequence elements of the key AZ protein RIM1 influence condensation propensity in a cellular environment. We show that RIM1 can undergo phase separation in live HEK293T cells and primary cultured neurons. Furthermore, using a large number of deletion mutants, we systematically investigated the contribution of the RIM1 structural domains and IDRs to its propensity to undergo phase condensation. Our data indicate that while the full-length RIM1 exhibits the highest propensity to form droplets, all tested truncation mutants, except those lacking any IDRs or consisting solely of IDR2 and a single proline-rich motif (PRM), form droplets with distinct properties. These findings indicate that the IDR regions of RIM1 significantly contribute to the protein’s capacity to undergo phase separation. They further show that the sequences flanking these IDRs shape the propensity and properties of droplet formation. We furthermore demonstrate that for RIM1 the enrichment of the protein in droplets increases exponentially with the length of the fragments. However, this relationship is not uniform across all sequence elements.

## Results

### Full-length RIM1 forms condensate-like structures consistent with LLPS in live HEK293T cells

**Fig. 1:**
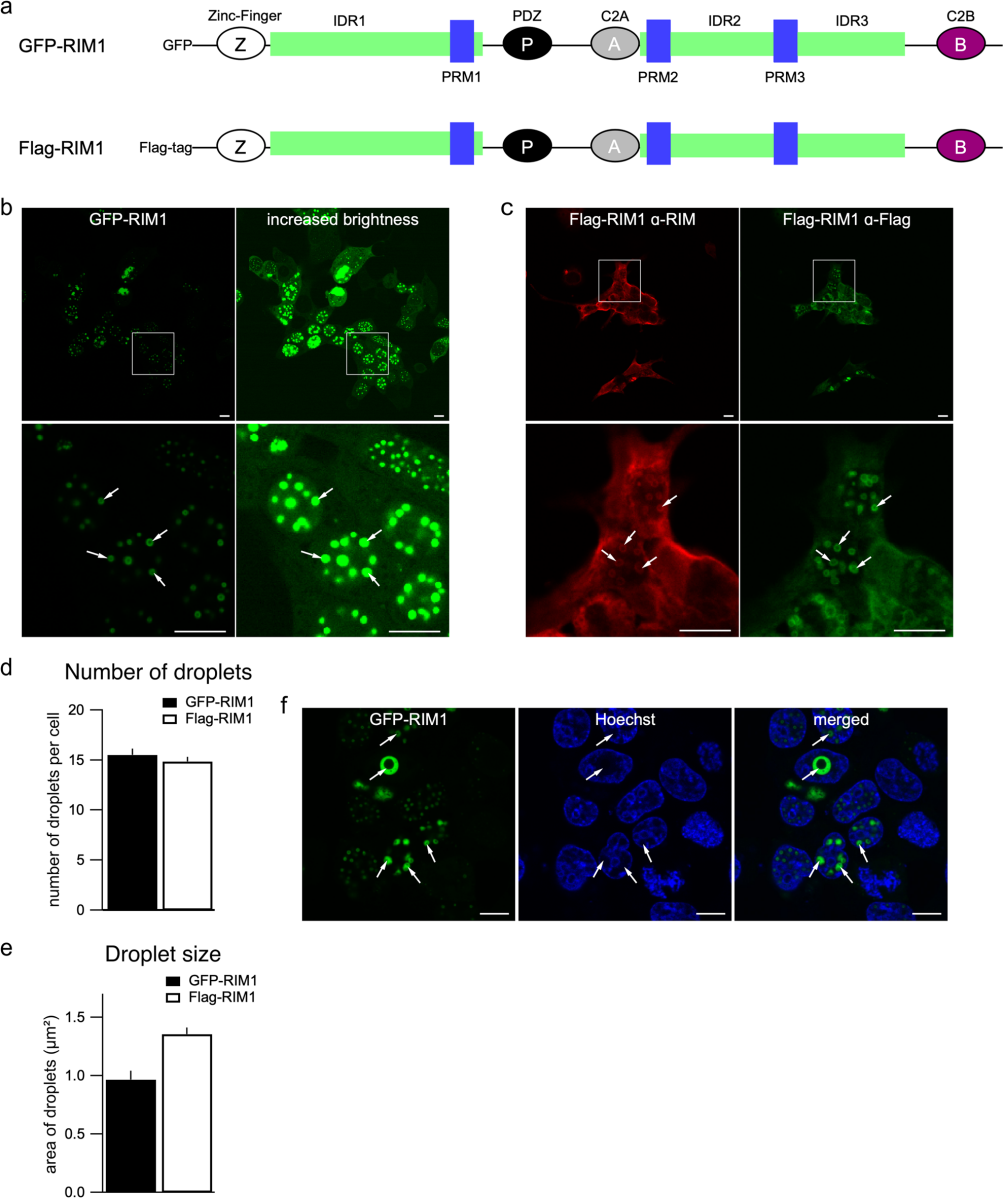
Full-length RIM1 forms droplets in transfected HEK293T cells independent of GFP- or Flag-tags. (**a**) Schematic representation of the domain structure of full-length RIM1 and the location of either enhanced GFP or the Flag-tag. (**b**) Representative single-plane confocal images of live HEK293T cells showing phase condensation of GFP-RIM1 in the nucleoplasm 24 h after transfection (arrows indicate phase condensates). (**c**) Representative single-plane confocal images of fixed HEK293T cells 24 h after transfection with Flag-RIM1, immunolabeled with an antibody against RIM1 and the Flag-tag (arrows indicate phase condensates). (**d**,**e**) Quantitative analysis of the number of RIM1 droplets per cell (GFP-RIM1: 15.52 ± 0.64, Flag-RIM1: 14.85 ± 0.47) and the size of droplets in µm^2^ (GFP-RIM1: 0.97 ± 0.08 µm^2^, Flag-RIM1: 1.35 ± 0.06 µm^2^; all quantitative data presented in the figures are provided in Suppl. Table S3) in HEK293T cells transfected with either GFP-RIM1 or Flag-RIM1; 3 independent experiments, 272 (GFP-RIM1) and 519 (Flag-RIM1) cells analyzed per construct (Mann-Whitney test, *p < 0.05). (**f**) Representative single-plane confocal images of live HEK293T cells transfected with GFP-RIM1 after Hoechst staining showing no overlap of DNA structures with the RIM1 droplets (arrows indicate phase condensates localized in regions lacking prominent Hoechst staining); scale bars = 10 μm.

Confocal imaging of live cells revealed the presence of GFP-RIM1 droplets in the nucleoplasm of the cells 24 h after transfection (Fig. 1b, Suppl. Fig. S2). The full-length RIM1 (FL-RIM1) N-terminally tagged with GFP (Fig. 1a) has been previously shown to be functionally equivalent to wild-type RIM1 in a synaptic release assay in hippocampal neurons^31^. When low DNA amounts of GFP-RIM1 were used (0.5, 1, or 10 ng/well), less than 25% of transfected HEK293T cells showed RIM1 phase condensates (0.5 ng: 5.4 ± 4.1%; 1 ng: 16.2 ± 2%; 10 ng: 20.2 ± 4.4%, Suppl. Fig. S2; all quantitative data shown in the figures are provided in Suppl. Table S3). To exclude the possibility that droplet formation may be caused by the GFP-tag, we performed the same experiment with RIM1 fused N-terminally to the short Flag-tag sequence (Fig. 1a). Immunolabeling with antibodies against the Flag-tag and RIM1 also revealed droplets in the nucleoplasm (Fig. 1c). These results demonstrate that in vivo droplet formation depends solely on the RIM1 protein sequence.

Next, we quantitatively analyzed the number of droplets per cell and droplet size, using single-plane images, which were assumed to be representative of the entire droplet population (analysis approach shown in Suppl. Fig. S1). These two parameters were considered linked to the strength of the protein interactions in the droplet. This analysis revealed that increasing the amount of the transfected GFP-RIM1 DNA did affect the size of the RIM1 phase condensates, as well as their abundance per cell (Suppl. Fig. S2). In cells expressing either GFP-RIM1 or Flag-RIM1 following transfection with 500 ng of DNA per well, on average, 15 droplets per cell with a droplet cross-sectional area of 0.97 ± 0.08 (GFP) and 1.35 ± 0.06 µm^2^ (Flag) (Fig. 1d and e) were detected.

Since the RIM1 droplets were unexpectedly found in the nucleoplasm rather than the cytoplasm of the HEK293T cells, we next examined whether droplets were associated with nuclear DNA structures. To this end, we stained the HEK293T cells expressing GFP-RIM1 with the DNA-binding dye Hoechst 33342 (Fig. 1f). No overlap between the DNA structures and the GFP-RIM1 droplets was observed, supporting the idea that the droplets representing condensate-like structures consistent with LLPS in the nucleus are independent of nuclear DNA.

**Fig. 2:**
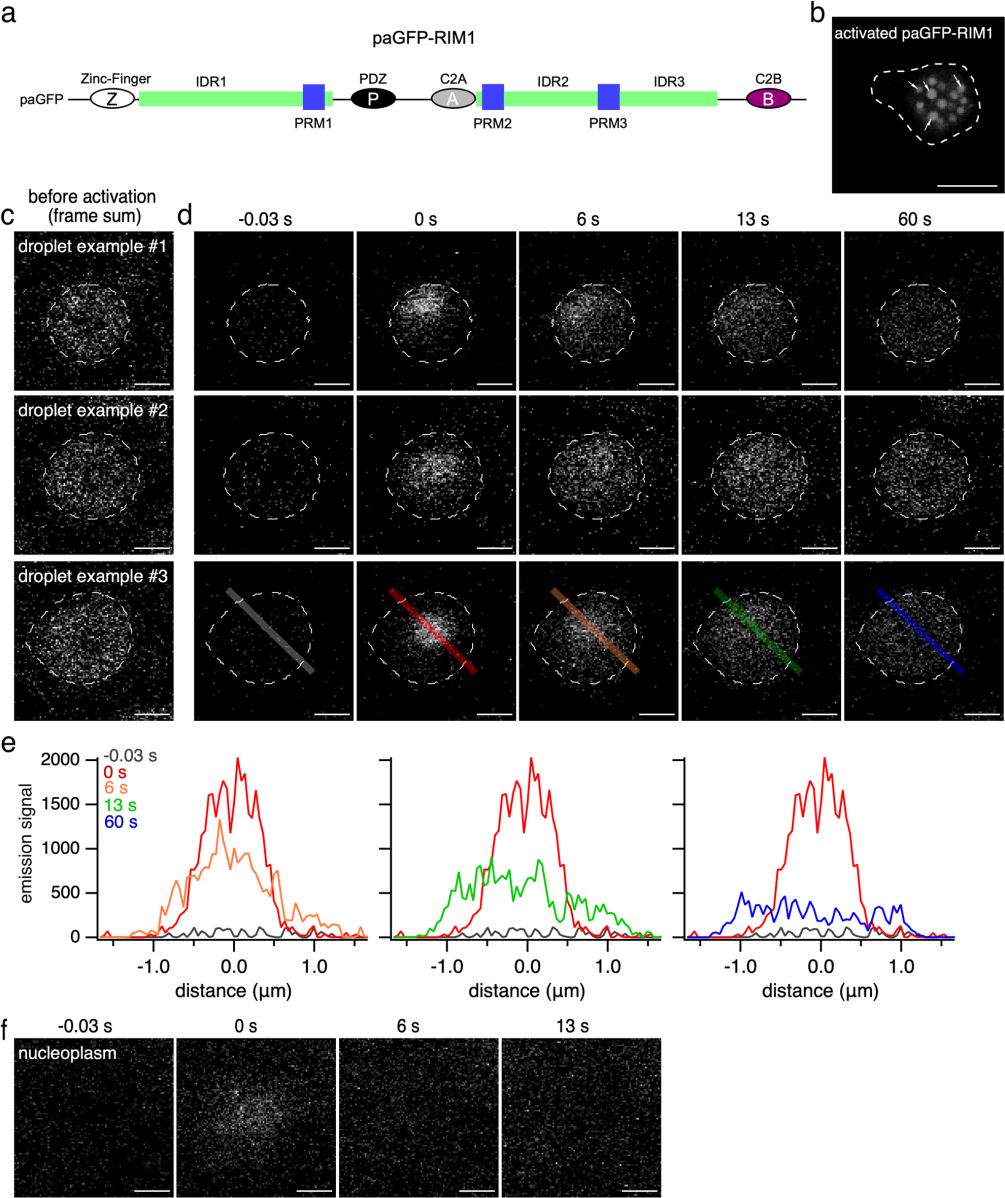
In the nucleus of transfected HEK293T cells, full-length RIM1 forms droplets exhibiting liquid-like properties consistent with liquid-liquid phase separation. (**a**) Schematic representation of the domain structure of full-length RIM1 and the location of photoactivatable GFP-tag (paGFP-RIM1). (**b**) Representative single-plane image of a live HEK293T cell after whole-cell photoactivation showing paGFP-RIM1 condensates in the nucleoplasm 24 h after transfection (cell boundaries are outlined by a dashed line, arrows indicate paGFP-RIM1 condensates). (**c**) Representative examples of paGFP-RIM1 droplets in different live HEK293T cells before activation, depicted as the sum of 10 pre-activation frames, confirming that the subsequent local point photoactivation was performed within individual condensates. (**d**) Representative frames from time-lapse imaging of paGFP-RIM1 droplets from (**c**) after local point photoactivation within condensates (activation at 0 s), showing the spread of activated paGFP-RIM1 throughout the droplets over time (droplet boundaries are outlined by dashed lines in (**c**,**d**)). (**e**) Emission line profiles from droplet example #3 at the time points shown in (**d**), illustrating spatial redistribution of the activated paGFP-RIM1 signal. Note that the fluorescence signal rapidly spreads within the droplet but remains confined to it for many seconds (15 droplets were analyzed and showed similar behavior). (**f**) Rapid dissipation of the activated paGFP-RIM1 in the nucleoplasm without its spatial confinement; scale bars = 10 μm (**b**) and 1 μm (**c**,**d**,**f**).

To determine whether RIM1 forms condensates exhibiting liquid-like properties in living cells, we transfected HEK293T cells with a plasmid encoding FL-RIM1 fused to photoactivatable GFP (paGFP-RIM1, Fig. 2a). Whole-cell photoactivation revealed the formation of paGFP-RIM1 droplets in the nucleus of cells (Fig. 2b). Point-like photoactivation within droplets was then used to assess the mobility of material within condensates. Following point activation of paGFP-RIM1 within individual condensates, the signal progressively spread throughout the droplets over time while remaining spatially confined within droplet boundaries for several seconds (Fig. 2c-e, 15 droplets were analyzed). In contrast, photoactivated paGFP-RIM1 in the nucleoplasm, outside of droplets, rapidly dispersed without spatial confinement (Fig. 2f). These observations demonstrate that RIM1 molecules remain mobile within condensates and show a reduced exchange with the bulk solution, supporting their formation via LLPS.

Taken together, our results show that FL-RIM1 forms droplets in the nucleus of live HEK293T cells, indicating, in accordance with previous studies^16,19–21,42,43^, the formation of concentration-dependent, spherical, microscopically resolvable condensate-like structures consistent with phase separation behavior.

### Full-length RIM1 forms droplets in neurons

**Fig. 3:**
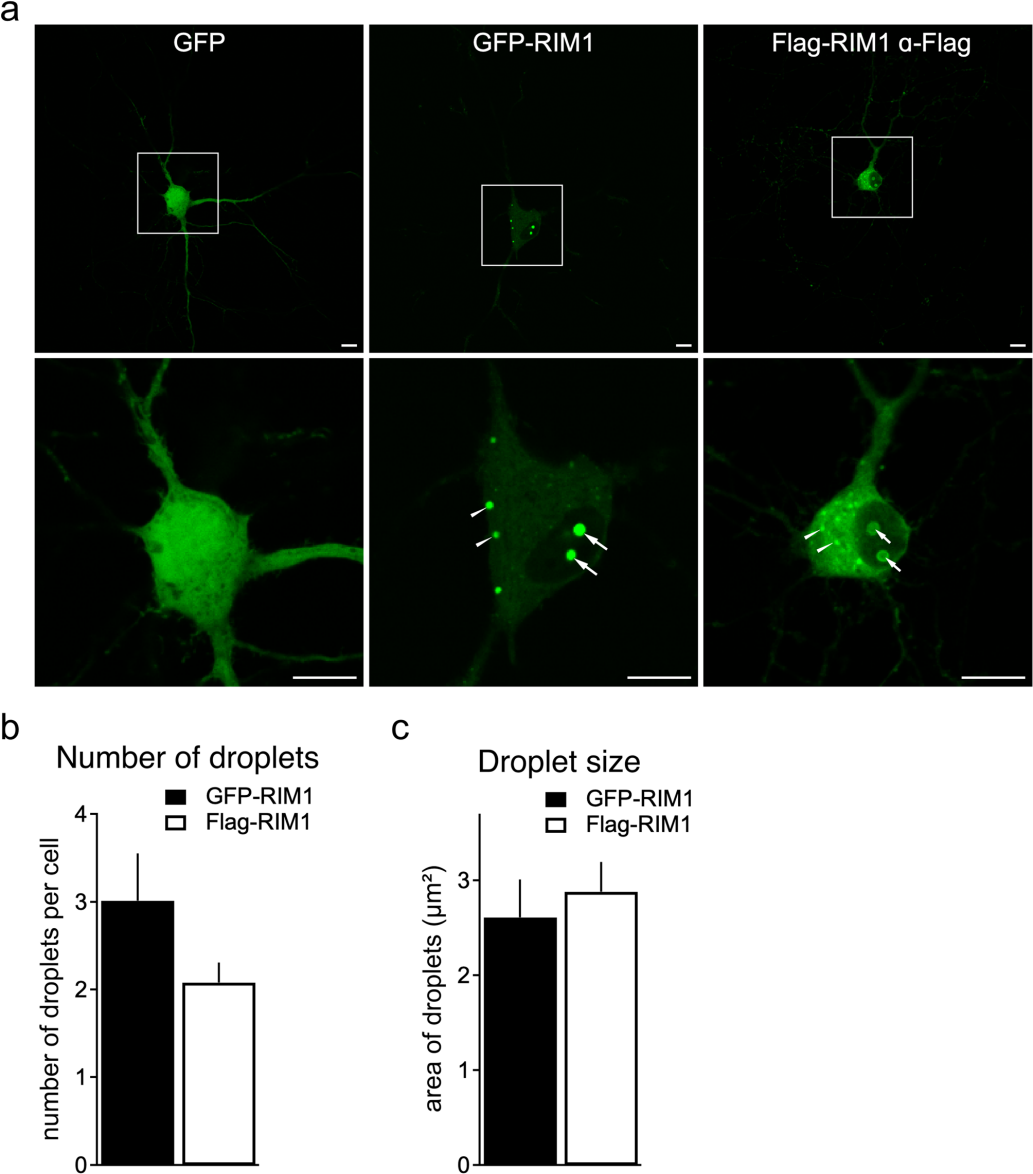
Full-length RIM1 undergoes phase separation in the nucleus and the cytoplasm of primary hippocampal neurons. (**a**) Representative single-plane confocal images of primary hippocampal neurons transfected with GFP or GFP-RIM1 (live cells) and Flag-RIM1 (cells were fixed and subsequently immunolabeled against the Flag-tag). Condensates formed by GFP-RIM1 and Flag-RIM1 in the nucleus are marked by arrows, and those in the cytoplasm by arrowheads. (**b**,**c**) Bar graphs showing the quantitative analysis of the number of droplets formed by Flag- and GFP-RIM1 in hippocampal neurons (GFP-RIM1: 3.01 ± 0.54, Flag-RIM1: 2.08 ± 0.23) and their area in µm^2^ (GFP-RIM1: 2.61 ± 0.4 µm^2^, Flag-RIM1: 2.88 ± 0.31 µm^2^); 3 independent experiments, 49 (GFP-RIM1) and 104 (Flag-RIM1) cells analyzed per construct (Mann-Whitney test, *p < 0.05); scale bars = 10 μm.

We next examined whether RIM1 droplets can also be observed in neurons. To this end, we expressed full-length RIM1 in primary hippocampal neurons. Confocal imaging of the neurons 10 days after transfection with GFP-RIM1 revealed droplets in the somatic cytoplasm and the nucleus of the neurons, whereas GFP alone exhibited a uniform distribution throughout the soma (Fig. 3a). The same result was obtained when we expressed Flag-RIM1 (Fig. 3a), indicating that also in neurons droplet formation depends on the RIM1 protein sequence and not on the tag. Quantification of the droplet number in neurons expressing either GFP-RIM1 or Flag-RIM1 revealed an average of three and two droplets per cell, respectively (Fig. 3b). The average area of the droplets was approximately two-fold larger in neurons (~ 2.5 µm^2^, Fig. 3c) than in HEK293T cells (Fig. 1e) for both constructs. In summary, these data show that FL-RIM1 constructs also form droplets in neurons.

### The proline-rich motifs (PRMs) 2 and 3 are not required for droplet formation of full-length RIM1

**Fig. 4:**
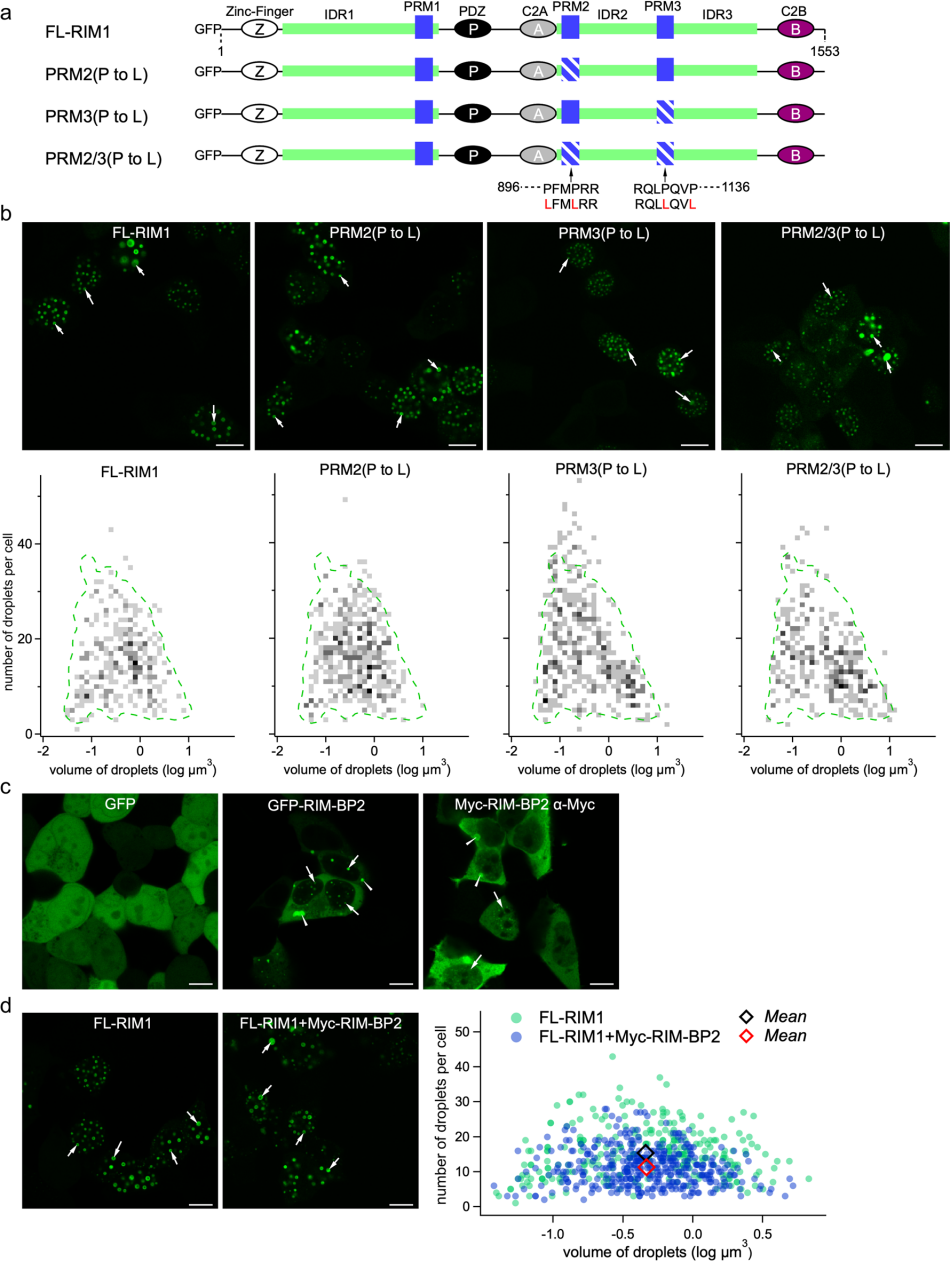
SH3-domain binding capacity of PRM2 and PRM3 is not required for RIM1 phase condensation. (**a**) Schematic representation of the composition of full-length RIM1 and of RIM1 mutants, in which the SH3-domain binding motifs, PRM2 and PRM3, were mutated by exchanging two prolines (P) to leucines (L), either separately or in combination (all constructs are fused to enhanced GFP); numbers indicate amino acid positions. (**b**) Representative single-plane confocal images of live HEK293T cells 24 h after transfection, showing that RIM1 droplet formation does not require functional PRM2 and PRM3 (arrows mark phase condensates) and plots showing the distribution of cells as a function of mean droplet volume (log_10_-transformed values) and droplet number per cell for RIM1 and the mutants PRM2(P to L), PRM3(P to L), and PRM2/3(P to L). Pixel intensity is depicted relative to the number of cells containing droplets of a given mean volume and number, ranging from 0 (white) to 4–5 (black) cells, green dashed line delineates distribution observed for FL-RIM1, generated from a pooled dataset of wild-type RIM1 and its point mutants. (**c**) Representative single-plane confocal images of HEK293T cells transfected with GFP or GFP-RIM-BP2 (live cells) and Myc-RIM-BP2 (cells were fixed and subsequently immunolabeled against the Myc-tag), showing that, in contrast to GFP, RIM-BP2 forms detectable droplets autonomously in both the nucleus and cytoplasm (nuclear condensates are indicated with arrows and cytoplasmic condensates with arrowheads). (**d**) Representative single-plane confocal images of live HEK293T cells transfected with GFP-RIM1 alone, or co-transfected with GFP-RIM1 and Myc-RIM-BP2 (arrows mark phase condensates), and scatter plot of cell distribution as a function of mean droplet volume (log_10_-transformed values) and droplet number per cell for GFP-RIM1-transfected and GFP-RIM1/Myc-RIM-BP2-co-transfected cells, indicating that RIM-BP2 does not affect FL-RIM1 droplet properties; 3 independent experiments and 4 images per experiment, total number of cells analyzed per construct: RIM1: 272, PRM2(P to L): 388, PRM3(P to L): 370, PRM2/3(P to L): 292, RIM1 without/with RIM-BP2: 374/405; scale bars = 10 μm.

Our results so far show that RIM1 forms droplets in HEK293T cells and primary neurons. Experiments with purified RIM1 fragments in cell-free assays showed that the region from amino acid (AA) 871 to 1097, which contains two proline-rich motifs (PRMs), PRM2 and PRM3, that mediate the interaction of RIM1 with RIM-BP, is sufficient to form phase-separated condensates in the presence of RIM-BP^15^. However, it is still unresolved whether this region of RIM1 is sufficient or required to induce phase condensation of RIM1 in a cellular environment. Therefore, we next tested if the ability of the PRMs to bind to SH3-domain proteins or other sequence motifs is required in this process in the cellular context of HEK293T cells. It is well established that the core prolines in PRM sequences are required for their interactions with SH3-domains and that mutating them interferes with binding^44–50^. Therefore, we mutated the proline (P) residues in PRM2 (**P**FM**P**RR**)** and PRM3 (RQL**P**QV**P)** to leucine (L) in FL-RIM1 (Suppl. Data S1) to abolish binding to SH3-domains, and tested the impact of mutations in either PRM alone or in combination (Fig. 4a).

Individual HEK293T cells expressing the same construct showed a characteristic degree of variability for droplet number and mean droplet volume. To illustrate this variability and the frequency of HEK293T cells showing a certain combination of these two parameters, we computed a 2D histogram for each construct (Fig. 4b). This analysis revealed no substantial difference in the distribution of droplet volume and abundance between FL-RIM1 and the mutants in which the prolines in PRM2, PRM3, or PRM2/3 were mutated. Given the similarity of the distributions, cells transfected with wild-type FL-RIM1 and all proline mutants were pooled to generate a robust “FL-RIM1” sample. A 2D histogram (bin width: 0.1 for log droplet volume and 1 for droplet number per cell) was then constructed from the “FL-RIM1” dataset and smoothed using a Gaussian filter (kernel size = 5×5 bins, corresponding to the Gaussian filter width of σ = 0.1227 for the log volume axis and σ = 1.227 for the droplet number axis) to reduce sampling noise. The high-probability region of the histogram was visualized using an isolevel contour line, with the level selected as 10% of the maximum bin count to best represent the overall shape of the distribution (Suppl. Fig. S3 illustrates the entire analysis workflow for contour generation). The resulting outline therefore shows the typical distribution of FL-RIM1 droplet properties across cells (dashed green line in Fig. 4b, lower panel) and is intended to capture the high-probability region rather than to include all individual data points. The PRM3(P to L) mutant showed one difference from the generated characteristic FL-RIM1 outline, a slightly broadened distribution pattern, shifted toward a larger number of droplets per cell. This indicates that in HEK293T cells FL-RIM1 PRMs 2 and 3 play only a minor role for droplet formation.

Next, we examined whether RIM-BP2, which in neurons binds to the RIM1 PRMs, is able to form droplets in HEK293T cells. In contrast to GFP, which was diffusely distributed throughout the cell, both GFP-RIM-BP2 and Myc-RIM-BP2 formed condensates in the nucleus and cytoplasm, indicating that RIM-BP2 is capable of autonomous phase separation and that condensate formation is driven by the intrinsic properties of the protein sequence rather than by the fusion tag (Fig. 4c and Suppl. Fig. S4). If GFP-RIM1 and Myc-RIM-BP2 were co-expressed, RIM1 droplet localization was not restricted to the nucleus and extended also into the cytoplasm (Suppl. Fig. S2). No pronounced effect of Myc-RIM-BP2 on the size or abundance of FL-RIM1 droplets was observed when 500 ng/well of GFP-RIM1 DNA was used for transfection, which presumably corresponds to a high intracellular RIM1 concentration (Fig. 4d). In summary, these findings show that in a cellular environment, RIM-BP2 forms droplets like RIM1 and does not impact the propensity of FL-RIM1 to undergo phase condensation or RIM1 droplet properties when RIM1 is present at a high intracellular concentration.

### RIM1 droplet formation does not require the zinc finger and C2B domains

**Fig. 5:**
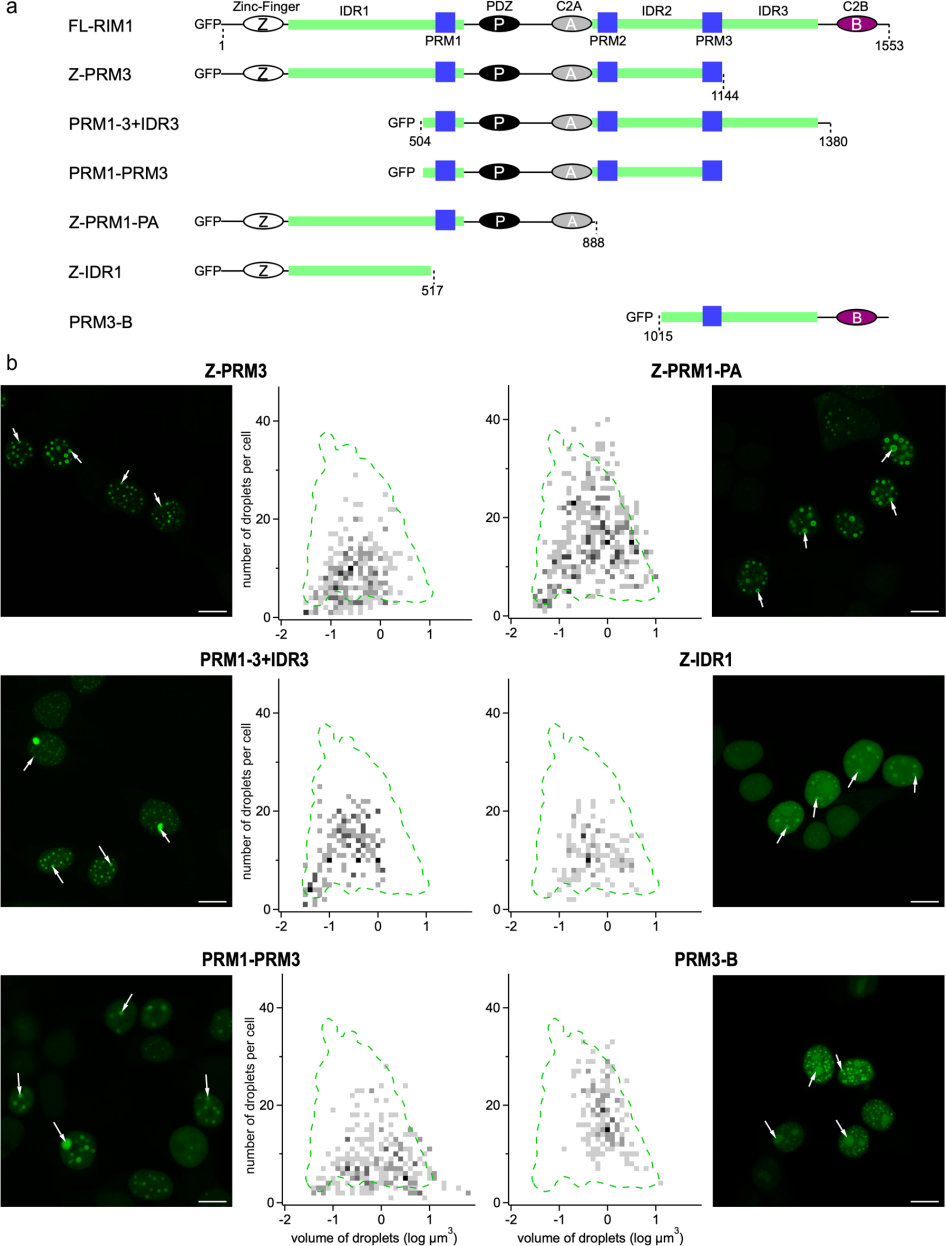
Droplet properties depend on the specific combination of IDRs and structural domains. (**a**) Schematic representation of the composition of full-length RIM1 and of the truncation mutants (all constructs are fused to enhanced GFP). (**b**) Representative single-plane confocal images of live HEK293T cells 24 h after transfection with the RIM1 truncation mutants shown in (**a**) (phase condensates are indicated by arrows) and plots showing the distribution of cells as a function of mean droplet volume (log_10_-transformed values) and droplet number per cell for Z-PRM3, PRM1-3 + IDR3, PRM1-PRM3, Z-PRM1-PA, Z-IDR1, and PRM3-B. The distribution observed for Z-PRM1-PA closely resembles that of FL-RIM1. Z-PRM3 and PRM1-3 + IDR3 display a shift toward cells with smaller droplets, whereas PRM1-PRM3 shows a tendency toward larger droplet volumes. Z-IDR1 exhibits a loss of cells with both small and large droplets. For Z-PRM3, PRM1-3 + IDR3, Z-IDR1, and PRM1-PRM3, cells containing more than 20 condensates were rare. PRM3-B forms a heterogeneous condensate population within individual cells (numerous small condensates and 1–3 large ones), resulting in a shift toward a larger mean droplet volume while maintaining a droplet number range comparable to FL-RIM1. Pixel intensity is depicted relative to the number of cells containing droplets of a given mean volume and number, ranging from 0 (white) to 4–5 (black) cells, green dashed line delineates distribution observed for FL-RIM1; 3 independent experiments and 4 images per experiment, total number of cells analyzed per construct: Z-PRM3: 253, PRM1-3 + IDR3: 129, PRM1-PRM3: 243, Z-PRM1-PA: 337, Z-IDR1: 120, PRM3-B: 146; scale bars = 10 μm.

Next, we asked if the sequences N- and C-terminal to the region flanked by the PRM1 and PRM2/PRM3 are necessary for droplet formation in living cells by generating a series of RIM1 truncation mutants: Z-PRM3 (AA 1–1144) is lacking the region C-terminal to PRM3 consisting of IDR3 and the C2B-domain, PRM1-3 + IDR3 (AA 504–1380) starts ~ 20 amino acids upstream of PRM1 and ends after IDR3 lacking the zinc finger and the C2B-domain, PRM1-PRM3 (AA 504–1144) is a further truncated derivative of PRM1-3 + IDR3 with IDR3 removed, Z-PRM1-PA (AA 1–888) consists of the N-terminal half of RIM1 ending after the C2A-domain, Z-IDR1 (AA 1–517) is composed of the RIM1 N-terminus, including the zinc finger and IDR1, and PRM3-B (AA 1015–1553) includes a part of IDR2, PRM3, IDR3 and the C2B-domain (Fig. 5a, Suppl. Data S1 and Suppl. Table S1). Confocal imaging of the live HEK293T cells revealed that all six truncation mutants were capable of droplet formation (Fig. 5b).

We computed 2D histograms for each construct across 120–330 HEK293T cells from 3 replicates, showing the distribution of cells as a function of droplet number and mean droplet volume (Fig. 5b, for comparison, the distribution of FL-RIM1 is delineated by the dashed green line). This analysis revealed that only the mutant Z-PRM1-PA lacking the sequence downstream of the C2A-domain exhibited a distribution similar to the one observed for FL-RIM1. The other RIM1 mutants exhibited shifts in their distribution pattern for volume and number of droplets. Lack of the sequence downstream of PRM3 (Z-PRM3) resulted in a shift toward cells with smaller droplets. A similar shift was also found if the sequences N-terminal to PRM1 and C-terminal to IDR3 were deleted (PRM1-3 + IDR3). Z-IDR1, which includes only the zinc finger domain and IDR1, demonstrated a loss of cells with both large and small droplets, whereas a shift toward cells with a larger droplet volume was observed for PRM1-PRM3, composed of all PRMs, two structural domains (PDZ and C2A), and IDR2. For the above-mentioned constructs (Z-PRM3, PRM1-3 + IDR3, Z-IDR1, and PRM1-PRM3), cells containing a high number of condensates (> 20) were rare. Notably, the RIM1 fragment consisting of a part of IDR2, PRM3, IDR3, and the C2B-domain (PRM3-B) showed a heterogeneous condensate population within each transfected cell, exhibiting a high number of small condensates and 1–3 large ones. This resulted in a shift of the cell distribution toward a larger mean droplet volume while maintaining a droplet number range comparable to FL-RIM1. These results show that in HEK293T cells, even RIM1 fragments with only one structural domain and one full-length IDR (PRM3-B and Z-IDR1) can form droplets. However, these data indicate that the size of the droplets and their abundance in cells depend on the specific domains and sequences present in the fragments.

### RIM1 propensity to undergo phase separation requires at least one IDR

**Fig. 6:**
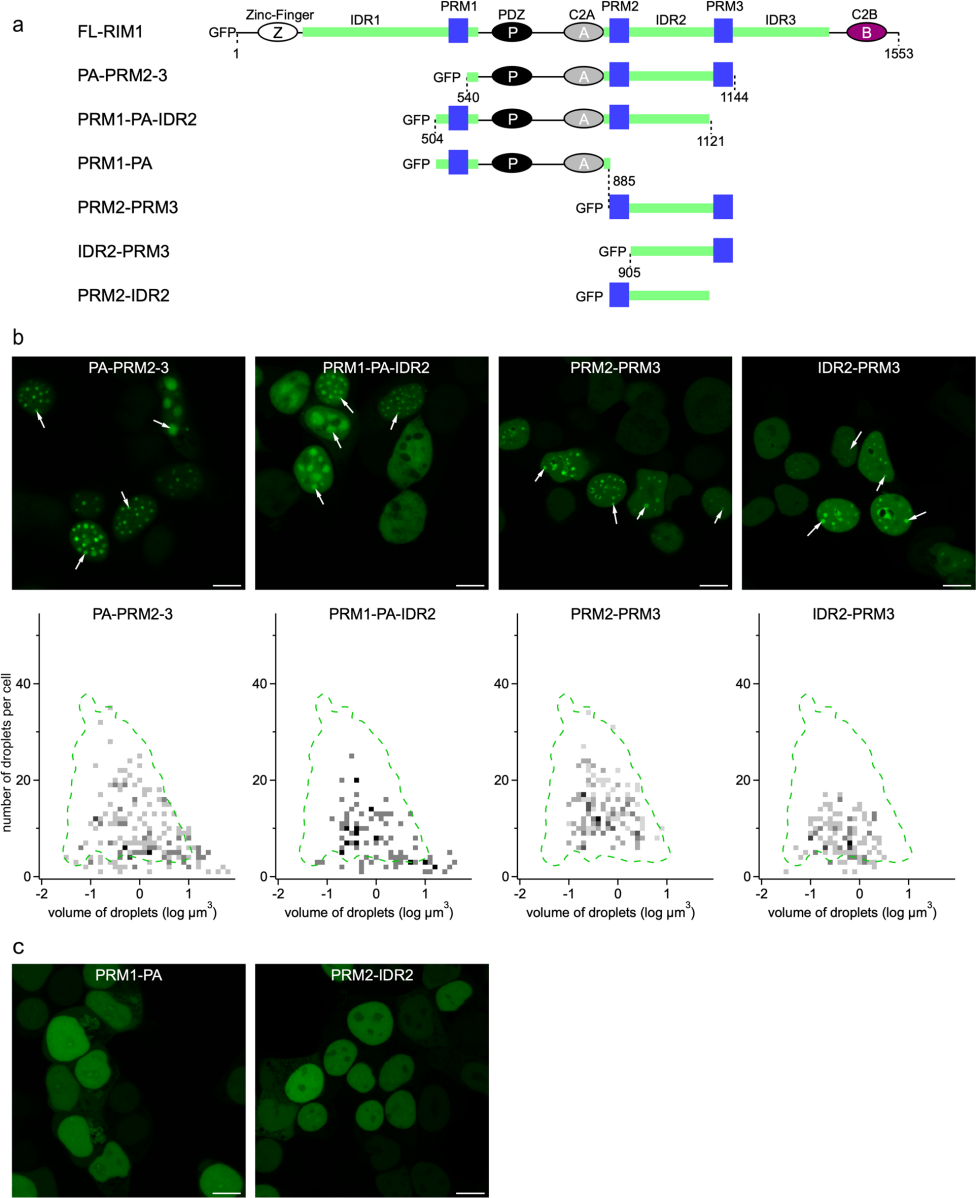
The central region of RIM1 without an IDR and PRM2 + IDR2 are not able to undergo phase condensation. (**a**) Schematic representation of the structure of full-length RIM1 and RIM1 truncation mutants containing at least one IDR or PRM (all constructs are fused to enhanced GFP). (**b**) Representative confocal images of live HEK293T cells 24 h after transfection with the RIM1 truncation mutants depicted in (**a**) (phase condensates are indicated by arrows) and plots showing the cell distribution between mean droplet volume (log_10_-transformed values) and droplet number per cell for PA-PRM2-3, PRM1-PA-IDR2, PRM2-PRM3, and IDR2-PRM3. PA-PRM2-3 and PRM1-PA-IDR2, both of which contain one IDR and two PRMs, exhibit a cell distribution shifted toward larger droplet sizes and a reduced number of droplets per cell relative to FL-RIM1. For PRM2-PRM3 and IDR2-PRM3, reduced variability in condensate size and number across cells is observed. Pixel intensity is depicted relative to the number of cells containing droplets of a given mean volume and number, ranging from 0 (white) to 2 cells for PRM1-PA-IDR2 and 4–5 cells for the remaining constructs (black), green dashed line delineates distribution observed for FL-RIM1; 3 independent experiments and 4 images per experiment, total number of cells analyzed per construct: PA-PRM2-3: 189, PRM1-PA-IDR2: 87, PRM2-PRM3: 198, IDR2-PRM3: 117. (**c**) Representative confocal images of live HEK293T cells 24 h after transfection with PRM1-PA and PRM2-IDR2, which did not undergo phase condensation (plots are therefore not shown); scale bars = 10 μm.

We next aimed to determine the importance of the individual PRMs and adjacent regions for phase condensation. To this end, we generated GFP-RIM1 truncation variants in which either one or two PRMs had been deleted (Fig. 6a, Suppl. Data S1 and Suppl. Table S1). The 2D distribution of cells across droplet number and mean droplet volume was similar for the two fragments that contained the C2A- and PDZ-domain, two PRMs and IDR2 (PA-PRM2-3 and PRM1-PA-IDR2), which exhibited a shift toward cells with larger droplet volumes, and for the two mutants that were composed of IDR2 plus PRM2 and PRM3 or IDR2 plus PRM3 alone (PRM2-PRM3 and IDR2-PRM3, respectively), in which the droplet volume in the cell population was distributed around the average of FL-RIM with reduced variability. In addition, a tendency toward a lower number of droplets per cell was observed for PA-PRM2-3, PRM1-PA-IDR2, and IDR2-PRM3 compared with FL-RIM1 (Fig. 6b).

The most striking finding of this experiment was that no clearly visible droplets in sufficient numbers were observed in confocal images of live HEK293T cells transfected with RIM1 truncation mutants consisting of either only the RIM1 central region from PRM1 to the end of the C2A-domain or of PRM2 and IDR2 (PRM1-PA and PRM2-IDR2, respectively; Fig. 6c). For these RIM1 fragments, GFP fluorescence was uniformly distributed but still restricted mainly to the nucleus. These findings indicate that the RIM1 fragment without an IDR is unable to undergo phase separation (PRM1-PA) and that interactions mediated by PRM2 alone are not sufficient to support droplet formation in combination with one IDR (PRM2-IDR2).

### In HEK293T cells, RIM-BP2 does not promote droplet formation of full-length RIM1 but differentially impacts fragments containing PRM1 or PRM2/3

A previous study with purified RIM1 protein showed that at physiological salt concentrations (150–200 mM), the addition of a purified RIM-BP2 fragment containing three SH3-domains promotes phase separation of RIM1^15^. Our results demonstrate that in live cells, FL-RIM1 has the ability to form droplets if two of the RIM-BP2 binding motifs are either mutated (PRM2/3(P to L)) or absent (Z-PRM1-PA, PRM3-B, and IDR2-PRM3). To probe if the presence of RIM-BP2 has an impact on the propensity of FL-RIM1 to form droplets, we transfected HEK293T cells with increasing amount of GFP-RIM1 DNA (0.5, 1, 10, and 100 ng per well; in previous experiments 500 ng were used, when not specified explicitly), either alone or together with Myc-RIM-BP2 DNA at a 1:1 molar ratio to the RIM1 construct (Suppl. Fig. S2). Whereas the highest FL-RIM1 DNA amount (100 ng/well) resulted in the majority of transfected cells forming droplets, the fraction of cells with droplets is less than 25% at 0.5 to 10 ng of RIM1 DNA. The presence of RIM-BP2 increased the propensity of cells to form droplets at all tested RIM1 DNA amounts, with the effect being most evident at 10 ng/well. Whereas at low RIM1 DNA input (0.5 and 1 ng), co-transfection of Myc-RIM-BP2 caused the formation of larger droplets, a less pronounced impact on droplet size was observed at 10 ng and above. The number of droplets per cell was not significantly affected by the presence of RIM-BP2. As previously mentioned, we observed that in the presence of RIM-BP2, droplets also formed in the cytoplasm and not exclusively in the nucleus (Suppl. Fig. S2).

**Fig. 7:**
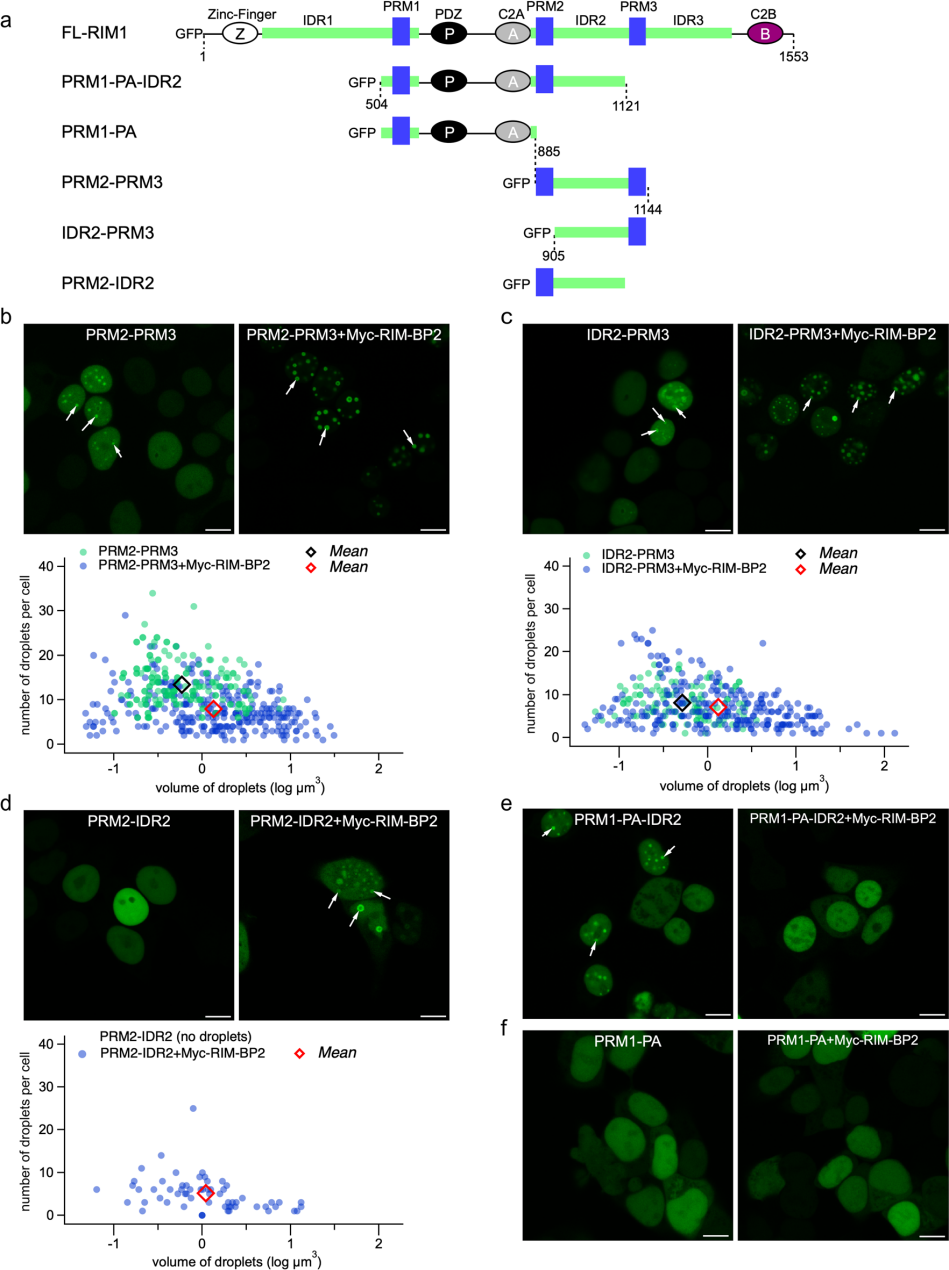
RIM-BP2 differentially modulates phase condensation of RIM1 truncation mutants in live HEK293T cells. (**a**) Schematic representation of FL-RIM1 and RIM1 truncation mutants (fused to enhanced GFP) co-transfected with Myc-RIM-BP2. (**b**–**d**) Representative confocal images of live HEK293T cells 24 h after co-transfection with Myc-RIM-BP2 and RIM1 truncation mutants containing at least one PRM of RIM1 that binds to RIM-BP2 (arrows indicate phase condensates) and scatter plots showing the distribution of cells as a function of mean droplet volume (log_10_-transformed values) and the number of droplets per cell (blue dots represent RIM1 truncation mutant co-transfected with Myc-RIM-BP2, and green dots those without Myc-RIM-BP2). The presence of RIM-BP2 increases droplet volume for PRM2-PRM3 and IDR2-PRM3, while reducing the number of droplets per cell only for PRM2-PRM3. RIM-BP2 enables phase condensation of PRM2-IDR2, which does not form droplets on its own. (**e**,**f**) Representative confocal images of live HEK293T cells 24 h after co-transfection with Myc-RIM-BP2 and RIM1 truncation mutants showing that RIM-BP2 suppressed droplet formation of PRM1-PA-IDR2 (arrows indicate phase condensates) and was not able to induce droplet formation for PRM1-PA; 3 independent experiments and 4 images per experiment, total number of cells analyzed per construct (without/with RIM-BP2): PRM2-PRM3: 198/392, IDR2-PRM3: 117/315, PRM2-IDR2: 0/59, PRM1-PA-IDR2: 87/0; scale bars = 10 μm.

We next tested whether co-transfection with Myc-RIM-BP2 exerts differential effects on phase separation capabilities of RIM1 truncation variants containing at least one PRM (Fig. 7a). For the two RIM1 fragments containing either PRM2 and PRM3 and the intermittent IDR2 (PRM2-PRM3, Fig. 7b) or PRM3 individually with the IDR2 (IDR2-PRM3, Fig. 7c), RIM-BP2 strongly increased the number of cells with a larger volume of droplets. The number of droplets per individual cell was decreased in the presence of RIM-BP2 for PRM2-PRM3 but unchanged for IDR2-PRM3 (Fig. 7b and c). Strikingly, RIM-BP2 induced phase condensation of the PRM2-IDR2 fragment (Fig. 7d), a truncation that lacked autonomous droplet-forming capacity, while fully suppressing phase separation of the PRM1-PA-IDR2 RIM1 fragment (Fig. 7e). In contrast, RIM-BP2 failed to promote condensation of the PRM1-PA fragment, which lacks an IDR and showed no baseline phase separation activity (Fig. 7f). Taken together, RIM-BP2 modulates RIM1 phase condensation, promoting droplet formation at low to intermediate RIM1 intracellular concentrations, while having no detectable stimulatory effect at higher RIM1 levels. RIM-BP2 increases the propensity of shorter PRM2/PRM3 containing RIM1 fragments to undergo phase separation and affects the droplet properties. In contrast, RIM-BP2 either does not induce or negatively impacts droplet formation in RIM1 fragments containing PRM1.

### Translocation of RIM1 to the nucleus is mediated by nuclear localization signals

**Fig. 8:**
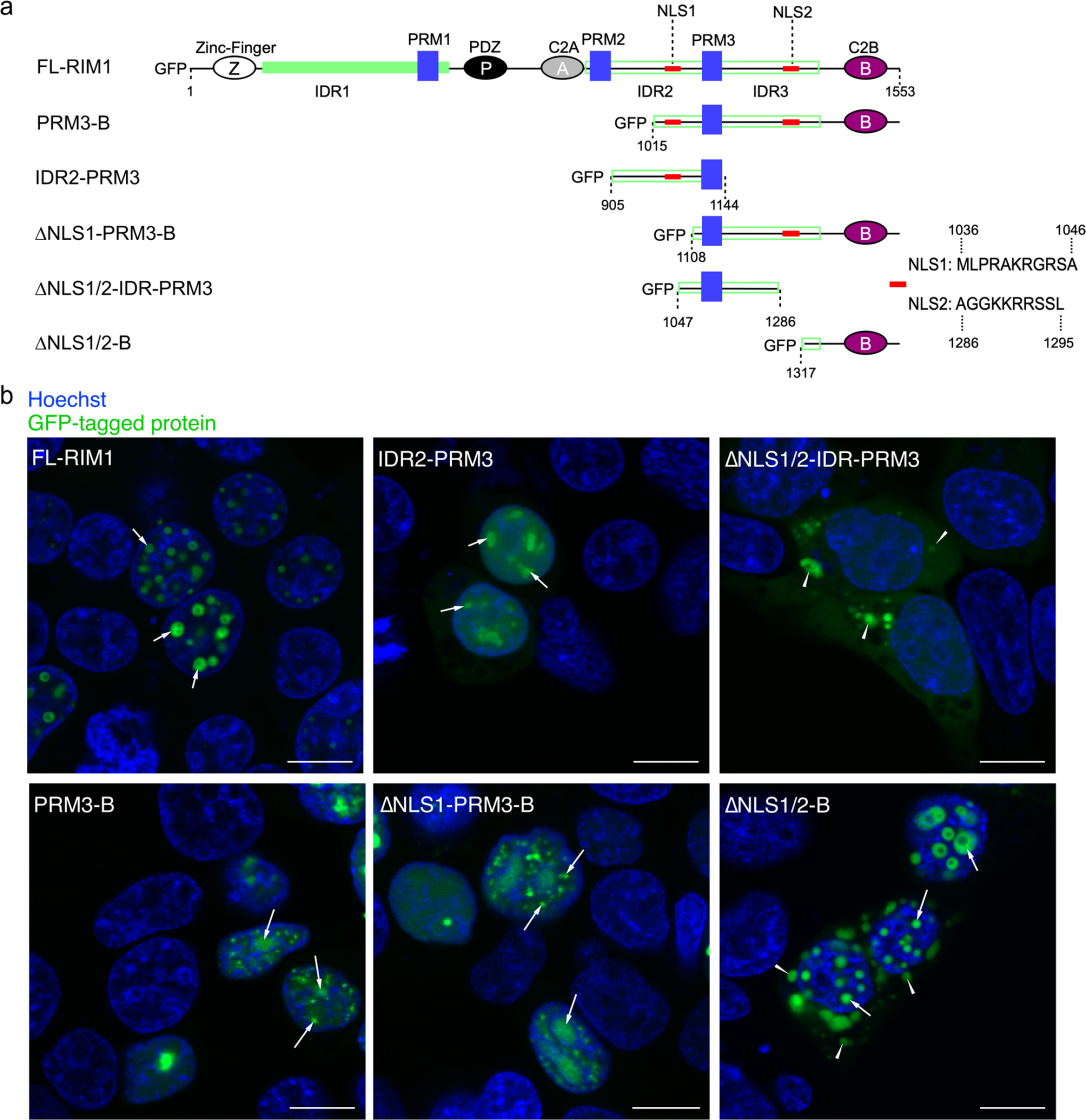
Translocation of RIM1 to the nucleus is mediated by nuclear localization signals. (**a**) Schematic representation of the truncation mutants used to test the function of bioinformatically predicted nuclear localization signals (NLS1 and NLS2, red boxes) in the C-terminal region of RIM1 (all constructs are fused to enhanced GFP). (**b**) Representative confocal images of live Hoechst-stained HEK293T cells 24 h after transfection with the truncation mutants depicted in (**a**), showing merged Hoechst and GFP signals. PRM3-B, containing both NLS1 and NLS2, localizes to the nucleus, confirming the functional activity of the predicted NLSs. Constructs containing only a single NLS (IDR2-PRM3 with NLS1 or ΔNLS1-PRM3-B with NLS2) also form nuclear condensates, demonstrating that a single NLS is sufficient for nuclear targeting. ΔNLS1/2-IDR-PRM3, lacking both NLS motifs, forms condensates exclusively in the cytoplasm, while ΔNLS1/2-B, a fragment comprising C2B-domain and its flanking regions but lacking NLS1 and NLS2, forms condensates in both the nucleus and cytoplasm, suggesting the presence of additional noncanonical NLSs within this fragment (nuclear condensates are indicated with arrows and cytoplasmic condensates with arrowheads); 3 independent experiments and 4 images per experiment, 90–150 cells per construct were inspected; scale bars = 10 μm.

One unexpected finding of our experiments so far was the observation that RIM1 droplets were present in the nucleus of both HEK293T cells and primary neurons (Figs. 1 and 3). A bioinformatic analysis of the RIM1 sequence using cNLS Mapper^51,52^ and NLSdb^53^ identified several potential mono- and bipartite importin α-dependent nuclear localization signals (NLSs) distributed throughout RIM1. We focused on two predicted monopartite NLSs located in the C-terminal region of RIM1: NLS1 (AA 1036–1046: MLPRAKRGRSA) in the IDR2 between PRM2 and PRM3, and NLS2 (AA 1286–1295: AGGKKRRSSL) in the IDR3 between PRM3 and the C2B-domain (Fig. 8a, Suppl. Data S1). To examine if these NLSs were indeed involved in the targeting of RIM1 to the nucleus, we tested truncated variants of the C-terminal part of RIM1 containing both NLSs (PRM3-B), only NLS1 (IDR2-PRM3), only NLS2 (∆NLS1-PRM3-B), or neither NLS (∆NLS1/2-IDR-PRM3 and ∆NLS1/2-B) (Fig. 8a, Suppl. Data S1 and Suppl. Table S1).

PRM3-B, which contains both NLSs, localized to the nucleus of HEK293T cells, supporting the relevance of the bioinformatically predicted NLS motifs. Notably, IDR2-PRM3 (containing only NLS1) and ΔNLS1-PRM3-B (containing only NLS2) also formed condensates in the nucleus, reconstituting the localization pattern of PRM3-B (Fig. 8b) and indicating that the presence of a single NLS is sufficient to direct RIM1 to the nucleus. In contrast, ΔNLS1/2-IDR-PRM3, which contains the PRM3-B region between NLS1 and NLS2 but lacks both motifs, formed droplets that were absent from the nucleus and found exclusively in the cytoplasm of HEK293T cells, whereas ΔNLS1/2-B, comprising the PRM3-B sequence downstream of NLS2 and including the C2B-domain, formed condensates in both the nucleus and the cytoplasm (Fig. 8b). This supports the idea that the localization of the C-terminal region of RIM1 in the nucleus is mainly due to the presence of the identified NLS1 and NLS2, which showed high nuclear targeting efficiency. The partial nuclear localization of ΔNLS1/2-B suggests the presence of additional noncanonical NLS motifs with reduced targeting efficiency within this fragment (AA 1318–1324: SGHKKLK and AA 1449–1459: IAKKKTRIARK), as predicted by the deep learning-based NLSExplorer tool^54^. In summary, these results show that RIM1 can be transported to the nucleus due to the presence of functional nuclear localization signals and that its propensity to form droplets is independent of its subcellular localization.

### Propensity of RIM1 to enter phase condensates correlates with sequence length

**Fig. 9:**
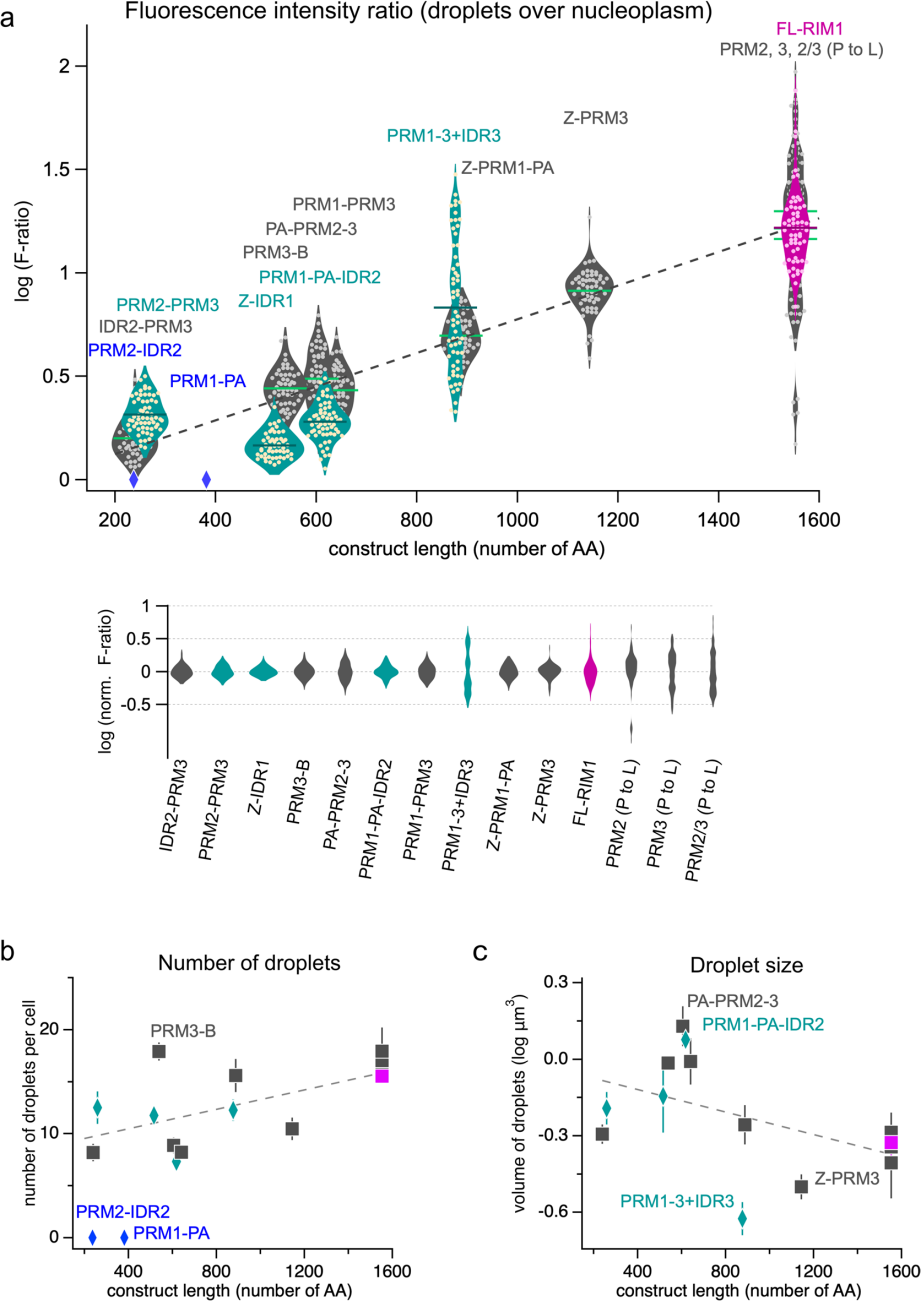
Sequence length is the main determinant for the propensity of RIM1 to form droplets. (**a**) Upper panel: Plot showing the correlation between the log_10_-transformed fluorescence intensity ratio (droplets/surrounding nucleoplasm, F-ratio) and protein sequence length for FL-RIM1 and mutants, calculated excluding GFP and linker sequences. The plot shows that the log of the F-ratio linearly correlates with the number of amino acids, except for PRM2-PRM3 and PRM1-3 + IDR3 (higher F-ratio), Z-IDR1 and PRM1-PA-IDR2 (lower F-ratio), and PRM2-IDR2 and PRM1-PA (no condensate formation). Lower panel: Graph depicting the distribution of the normalized log F-ratio for FL-RIM1 and mutants. The distribution is unimodal for all constructs except PRM1-3 + IDR3 and the three FL-RIM1 variants with mutated PRMs, which exhibit a broader, multimodal distribution of log F-ratio values; 3 independent experiments and 4 images per experiment, 60 cells per construct and 5 droplets per cell were analyzed. (**b**,**c**) Correlation between protein sequence length and droplet number (**b**) or log_10_-transformed droplet volume (**c**). A linear trend for more droplets per cell with increased AA number is observed, whereas the log droplet volume exhibits a scattered distribution not well described by a linear fit (constructs that deviate most from the linear trend are individually labeled); constructs failing to undergo phase separation were excluded from the linear fit, construct color coding: wild-type FL-RIM1 (magenta), constructs whose F-ratio deviates from the linear fit (green), constructs not undergoing phase separation (blue), and remaining constructs (gray).

To quantitatively assess the propensity of RIM1 for phase condensation, we determined the fluorescence intensity ratio between droplet and surrounding nucleoplasm (F-ratio, analysis approach shown in Suppl. Fig. S1) for all tested RIM1 truncation mutants and plotted it against the sequence length of the protein (Fig. 9a, upper panel). The fluorescence of the nucleoplasm approximates the concentration of the GFP-RIM1 protein variant remaining in the solution outside of the droplets, whereas the fluorescence of the droplets is related to the concentration of the protein variant in the droplets. A high F-ratio therefore means that in equilibrium, the protein is highly enriched in droplets and a low amount is found in a non-condensed form. The log of the F-ratio linearly correlates with the number of amino acids across all RIM1 variants (Fig. 9a, upper panel). Notably, there were six exceptions: PRM2-PRM3 and PRM1-3 + IDR3 showed a higher and Z-IDR1 and PRM1-PA-IDR2 a lower F-ratio than predicted for their length, and PRM2-IDR2 and PRM1-PA did not form droplets at all (Fig. 9a, upper panel, labeled green and blue). For most fragments, the distribution of the log F-ratio was fairly narrow and unimodal. This can be best seen in the lower panel of Fig. 9a. However, PRM1-3 + IDR3 and the three FL-RIM1 variants with the mutated PRMs exhibited a clearly broader and multimodal distribution of the log F-ratio values, indicating a higher variability of the propensity for phase condensation across cells.

The number of droplets per cell and the volume of droplets did not show a comparable dependence on construct length (Fig. 9b and c). While there was a linear trend for more droplets per cell with increased AA number (+ 50% across the set of constructs), the volume of the droplets exhibited a scattered distribution not well described by a linear fit.

## Discussion

Phase behavior of proteins is strongly influenced by the cellular environment, including salt concentration, temperature, pH, post-translational modifications, and the availability of binding partners^34,38,55^. It is therefore important to assess the contribution of individual protein domains not only in reconstituted, cell-free systems but also in living cells. Overexpression of synaptic proteins in heterologous cell lines including HEK293T cells has become a widely used assay to probe condensation propensity. In this setting, the appearance of microscopically resolvable droplets is used as a proxy for condensate formation and has been applied to numerous presynaptic proteins, including RIM1, RIM-BP, Liprin-α, Munc13, CASK, ELKS/ERC1 as well as postsynaptic scaffold proteins^16,19–21,42,43,56^. Our local photoactivation experiments showed that RIM1 molecules remain mobile within droplets formed in live HEK293T cells, supporting the interpretation of these condensates as LLPS-derived assemblies.

Phase condensation has been shown for protein fragments of most AZ proteins in vitro^15–18,57^ and condensate-like droplets have been observed after expression of fluorescently tagged RIM1, Munc13-1, and Liprin-α in HEK293T cells^20^. Against this background, the present study addresses a narrower but important question: which domains and sequence elements of RIM1 contribute to condensation in living cells? Using a systematic truncation and point-mutation approach in HEK293T cells and primary neurons, we identify three main findings. First, the propensity of RIM1 sequence elements to undergo phase condensation exhibits an unexpected strong exponential increase with protein length, indicating that the presence of all sequence elements is of critical significance to achieve a maximal degree of phase condensation. A few fragments deviate in their phase behavior from this general relationship (potential causes are discussed below). Second, fragments as short as ~ 250 amino acids can still form condensate-like assemblies in cells. Third, the contribution of individual motifs in the cellular context does not always match that inferred from purified fragments. Together, these results indicate that no single domain fully mimics the behavior of the full-length protein and that RIM1 condensation in cells is supported by sequence determinants distributed across the protein.

Our domain/sequence analysis showed that RIM1 does not require the full-length protein to form condensate-like assemblies in cells. Several truncated RIM1 sequences as short as around 250 amino acids formed droplets, which is smaller than the minimal regions reported so far for some other AZ proteins such as Liprin-α1 and ELKS, where phase separation requires substantially larger regions (600–1000 amino acids)^16,19,58^. Nevertheless, two fragments were unable to form droplets, PRM1-PA (PRM1, PDZ, C2A, and two linker sequences) and PRM2-IDR2. For PRM1-PA, the inability to undergo phase separation may be caused by the absence of an IDR, indicating that the structured regions alone are insufficient in this assay, and by the weak SH3-binding capacity of PRM1, which was previously demonstrated in vitro^15^, potentially due to the lack of a well-defined SH3-binding consensus motif. PRM2-IDR2’s failure to form phase condensates may be explained by the relatively weak contribution of IDR2, as predicted by MoRFchibi 2.0 (identifies molecular recognition features (MoRFs), short segments usually 10–79 residues)^59^, relative to IDR1 and IDR3, or by insufficient levels of PRM2-specific SH3-domain binding partners in HEK293T cells. A weak binding capability of PRM2 in the cellular context is supported by the finding that replacing PRM2 with PRM3 in an otherwise identical IDR2-containing construct (IDR2-PRM3) results in droplet formation, demonstrating that IDR2 itself is not inherently incompetent and that the identity of the flanking PRMs matters. This may reflect differences in SH3-binding class: PRM3 contains the class I-type core RQL**P**QV**P**, PRM2 the class II-type core **P**FM**P**RR^60^. These differences determine which SH3-domain proteins will interact and with what affinity.

This context dependence is further underscored by the discrepancy between cell-based and in vitro results. In the reconstituted system, the PRM-SH3 interactions between RIM1 and RIM-BP2 SH3-domains are key to condensate formation. Deletion of individual PRMs from a purified truncated RIM1 fragment strongly reduces LLPS, with PRM1 having the strongest effect despite its less well-defined SH3-binding core^15^. In contrast, in our cellular assay, individual deletion of PRM1 or PRM3 does not abolish droplet formation, and mutation of PRM2 and PRM3 in FL-RIM1 had little effect. The finding that individual PRMs in cells are not essential likely reflects the richer cellular environment, including post-translational modifications, molecular crowding, and a large number of potential interacting molecules, which collectively lower the threshold for condensate nucleation.

For most RIM1 fragments, phase condensation propensity, as estimated by the F-ratio, scales with sequence length, which is consistent with the distribution of protein-binding domains and sequence motifs across the protein. Four fragments deviate from this trend. PRM2-PRM3 and PRM1-3 + IDR3 phase-separate more than expected for their length. In these fragments multiple PRMs flank IDRs, which may enhance effective multivalency by enabling multiple simultaneous interactions. In contrast, Z-IDR1 and PRM1-PA-IDR2 form droplets less than predicted. For Z-IDR1, the zinc finger domain may act as a rigid spacer imposing steric constraints and its effective valency may be reduced as in HEK293T cells its neuronal binding partners, Munc13-1 and Rab3^61^, are either not expressed or at much lower levels than in neurons, respectively, based on data from the Human Protein Atlas^62^. PRM1-PA-IDR2 is composed of two sub-fragments, PRM1-PA and PRM2-IDR2, that on their own do not form droplets, and the combined sequence appears to be only weakly multivalent.

FL-RIM1 produces the highest and most consistent phase separation propensity, with most cells forming 10–20 droplets per cell with volumes around 0.5–0.8 µm^3^. The truncation mutants deviate from this pattern and fall into five distinct categories: (1) distribution similar to FL-RIM1 (Z-PRM1-PA), (2) absence or reduction of large droplets (PRM1-3 + IDR3, Z-PRM3), (3) shift toward larger droplet volume (PRM3-B), (4) fewer but larger droplets (PRM1-PRM3, PRM1-PA-IDR2, PA-PRM2-3), and (5) reduced droplet number with a loss of both small and large droplets (PRM2-PRM3, Z-IDR1, IDR2-PRM3). The general decrease in droplet number across the truncation mutants reflects fewer multivalent interactions resulting in a lower probability of condensate nucleation. Surprisingly, Z-PRM1-PA, which lacks the entire C-terminal half of RIM1 downstream of the C2A-domain, produces a distribution that is similar to FL-RIM1, whereas fragments consisting only of its N-terminal portion (Z-IDR1) or only its central structured region (PRM1-PA) deviate strongly from FL-RIM1. This finding demonstrates that the multivalency of a fragment is not simply the sum of individual sequence elements but that specific combinations of structural domains and sequence features can lead to an increased interaction capacity, possibly because of reduced steric hindrance or altered solvation properties^63^. Given that RIM1 shows extensive alternative splicing^1,64^, such combinatorial contributions of protein regions may be particularly relevant in a cellular context by impacting the subsynaptic localization of the protein. Three fragments that only differ slightly from each other, PRM1-PRM3, PRM1-PA-IDR2, and PA-PRM2-3, identify a minimal region of RIM1 consisting of the PDZ- and C2A-domain, IDR2, PRM2 and either PRM1 or PRM3 as sufficient to recapitulate the volume distribution of FL-RIM1. The appearance of the large droplet phenotype of PRM3-B may be driven by C2B-dimerization^65^, which increases effective valency in the absence of competing interaction domains^66^. The shortest fragments still capable of droplet formation, PRM2-PRM3, Z-IDR1, and IDR2-PRM3, show a strongly reduced cell-to-cell variability in droplet volume and number per cell compared to FL-RIM1. This is consistent with their simpler composition, which minimizes competing interactions and intramolecular folding^35^.

It has been reported that purified FL-RIM1 is monomeric at low concentrations, but can be induced to undergo LLPS by the presence of a RIM-BP2 fragment containing three PRM-binding SH3-domains^15^. According to the Human Protein Atlas^62^, none of the three RIM-BP proteins has been detected in HEK293T cells. Therefore, our data suggest that when RIM1 is overexpressed in HEK293T cells neither does FL-RIM1 require RIM-BP2 to form droplets nor does the mutation of PRM2 and PRM3, which bind to RIM-BP2, affect the properties of FL-RIM1 to undergo phase condensation. However, RIM-BP2 promotes droplet formation when cells are transfected with low amounts of FL-RIM1 DNA, which is expected to correspond to low intracellular RIM1 concentrations. As quantitative proteomics suggests that RIM1 is present at relatively low copy numbers in synaptic boutons^67^, RIM-BP2 may play a role in lowering RIM1’s condensation threshold under endogenous conditions. In addition, recent studies indicate that the properties of RIM1/RIM-BP condensates are strongly influenced by their molecular environment since lipid vesicles resembling synaptic vesicles stabilize the condensates in a liquid state and prevent their maturation into fibrillar aggregates^68^. This suggests that in vivo, the presynaptic lipid environment may further modulate RIM1/RIM-BP phase behavior and its localization dynamics.

RIM-BP2 also increases the propensity of short fragments containing IDR2 and PRM2/3 or PRM3 (PRM2-PRM3 and IDR2-PRM3 mutants) to undergo phase separation leading to an increase in the size of individual droplets, similar to that observed for FL-RIM1 at low intracellular concentrations. Strikingly, RIM-BP2 triggers condensate formation of a fragment, PRM2-IDR2, that is unable to form droplets on its own, indicating that the interaction of RIM-BP2 with domains of RIM1 (in this case, a PRM and an IDR) can promote the formation of a condensate by expanding network connectivity^23,24,69^. In contrast, RIM-BP2 negatively impacts PRM1-PA-IDR2, a fragment containing PRM1, suggesting that the interaction of RIM-BP2 with this fragment interferes with its multivalency.

Another finding of this study is the nuclear localization of RIM1 condensate-like assemblies under overexpression conditions in both HEK293T cells and primary neurons, which we show can be mediated by functional NLS motifs in the C-terminal half of the protein. The comparison between HEK293T cells and neurons suggests that RIM1 localization is further shaped by the cellular environment and available interaction partners. In HEK293T cells, overexpressed FL-RIM1 forms droplets predominantly in the nucleus, whereas co-transfection with RIM-BP2 leads to droplet formation in the cytoplasm as well. In transfected neurons, where RIM1 interaction partners are endogenously expressed, FL-RIM1 condensate-like assemblies are also observed in both the nucleus and the somatic cytoplasm. Similarly, our observation that RIM1 and RIM-BP2, but not other AZ proteins such as Liprin-α3, ELKS1αB, Munc13-1, and Bassoon (Suppl. Fig. S4), form droplets in the nucleus argues against nuclear localization being a generic property of all overexpressed AZ proteins. Although endogenous RIM1 has not been reported in the nucleus under basal conditions to date, future studies should examine whether RIM1 may have the capacity for regulated nuclear targeting and RIM1 has a potential physiological nuclear function.

We found that fragments containing the RIM1 zinc finger motif and IDR1 but lacking predicted NLSs (Z-IDR1 and Z-PRM1-PA) also formed droplets in the nucleus. Zinc finger domains in some proteins can interact with RNA^70,71^, and RNA is now well established as a component and modulator of many condensates^72^. Therefore, RIM1 might interact directly with RNA thereby influencing its phase condensation behavior and intracellular localization. This suggests that under physiological conditions, the propensity of FL-RIM1 to undergo phase condensation is enhanced, for example by post-translational modifications or the interaction with proteins and potentially also RNA.

As outlined above, we have applied an established overexpression approach in HEK293T cells for the systematic characterization of phase behavior of RIM1 structural domains and sequence elements. Though useful for a systematic comparison of constructs, it has certain limitations. First, the high protein levels resulting from overexpression may induce condensate formation in the absence of interactions or modifications that would be required under physiological conditions. Second, HEK293T cells do not express the endogenous RIM1 binding partners or mimic the local conditions and post-translational modifications of RIM1 that determine the threshold for RIM1 LLPS in neurons. We therefore view our results as the basis for defining candidate RIM1 regions that regulate condensation propensity in cells, not as a final model of how RIM1 phase behavior operates at synapses.

## Methods

### Animals and ethics statement

Primary hippocampal neurons were prepared from embryos of C57BL/6NCrl mice (Charles River, Germany). Animals were housed under a 12 h light/dark cycle, in a temperature-controlled (22 ± 2 °C) and humidity-controlled (55 ± 10%) environment with food and water available *ad libitum*. All animal procedures were planned and performed to minimize pain and suffering and to reduce the number of animals used in accordance with European, national, and institutional guidelines (European Directive 2010/63/EU, federal laws (TierSchG and TierSchVersV), and the guidelines of the Animal Care Committee of the University of Bonn Medical Centre). All experimental protocols were approved by the Landesamt für Natur, Umwelt und Klima (LANUK, North Rhine-Westphalia, Germany).

### Preparation and transfection of primary hippocampal neuronal cultures

Mouse hippocampal neurons were prepared from embryonic mice (E16–18) following a previously described procedure^31,73–75^. Briefly, hippocampi were removed from embryos, washed, and kept in ice-cold Hank’s balanced salt solution (HBSS) without calcium and magnesium (Gibco, Thermo Fisher Scientific). Tissues were digested with trypsin (0.25 mg/ml, without EDTA, Gibco, Thermo Fisher Scientific) for 20 min at 37 °C and then washed several times with HBSS. The remaining DNA was digested with DNase I (0.1 mg/ml, Roche). Cells dissociated by trituration were seeded into 24-well plates on glass coverslips (13 mm diameter, 0.13–0.16 mm thickness) coated with poly-D-lysine (Sigma-Aldrich) at a density of 2.5–3×10^4^ cells per well. Neurons were cultured in basal medium Eagle (BME, Gibco, Thermo Fisher Scientific) supplemented with 0.5 mM L-glutamine (Gibco, Thermo Fisher Scientific), 0.45% glucose (Sigma-Aldrich), 1% fetal bovine serum and 2% B-27 supplement (Gibco, Thermo Fisher Scientific). Cells were maintained at 37 °C and 5% CO_2_ in a humidified incubator until use.

Hippocampal neurons were transfected between 3 and 5 days in vitro (DIV) according to a previously described protocol^76^. Briefly, BME, in which the neurons were cultured, was removed shortly before transfection and stored for later use. Then, 0.5 ml/well of pre-warmed minimum essential medium (MEM, Gibco, Thermo Fisher Scientific) was added to each well. A transfection mixture containing 60 µl of 250 mM CaCl_2_, 65 µl of 2×BES-buffered saline (280 mM NaCl, 1.5 mM Na_2_HPO_4_, 50 mM BES, pH 7.15) and 2.5 µg of plasmid DNA was then added to each well. After 30 min of incubation at 37 °C and 2.5% CO_2_, the cells were washed with HBSS and returned to the previously collected original BME medium. Neurons were incubated at 37 °C and 5% CO_2_ before live cell imaging or fixation with 4% paraformaldehyde (PFA) for 15 min at room temperature (RT) at DIV 13–15.

### HEK293T cell culture and transfection

HEK293T (Cat. No. 632273, Takara Bio inc.) cells were cultured as described previously^74,75^. In brief, HEK293T cells were grown in Dulbecco’s modified Eagle’s medium (DMEM, Gibco, Thermo Fisher Scientific) supplemented with 1% penicillin-streptomycin and 10% fetal bovine serum (Gibco, Thermo Fisher Scientific) at 37 °C and 5% CO_2_ in a humidified incubator. Cells were seeded at a density of 4×10^4^ cells per well either on 13 mm diameter glass coverslips (0.13–0.16 mm thickness) placed in 24-well plates or in glass-bottom 24-well plates, all coated with poly-D-lysine, 24 h prior to transfection.

HEK293T cells were transfected with Lipofectamine 2000 (Invitrogen, Thermo Fisher Scientific) according to the manufacturer’s protocol. Briefly, the culture medium was replaced with Opti-MEM I reduced-serum medium (Gibco, Thermo Fisher Scientific) 30 min prior to transfection. For transfection, 500 ng of plasmid DNA per well was used. For co-transfection experiments, the amount of Myc-RIM-BP2 plasmid DNA was adjusted to a 1:1 molar ratio relative to the GFP-RIM1 plasmid (if needed, an empty vector plasmid was added to maintain a total DNA amount of at least 500 ng per well). Plasmid DNA was combined with 1 µl Lipofectamine 2000 per well. Cells were incubated at 37 °C in 5% CO_2_ for 24 h before live cell imaging (for experiments requiring DNA visualization, Hoechst 33342 (Invitrogen, Thermo Fisher Scientific) was applied to live HEK293T cells at a final concentration of 5 µg/ml for 10 min before imaging) or fixation with 4% PFA for 15 min at RT.

### Cloning and site-directed mutagenesis

The RIM1-derived plasmids used in this study were generated by the In-Fusion Cloning Kit (Takara Bio Inc.) according to the manufacturer’s protocol using the full-length rat RIM1 N-terminally fused to enhanced GFP in an expression plasmid under the control of the EF1α promoter (EF1α-EGFP-RIM1)^31^ as a template and backbone plasmid (the FL-RIM1 sequence is provided in Suppl. Data S1 (UniProt accession number: F1LYS1), and amino acid positions (start-end) of all RIM1 truncation mutants, numbered relative to the FL-RIM1 sequence, are listed in Suppl. Table S1). In brief, In-Fusion reactions were performed with the backbone linearized by the appropriate restriction enzymes and pre-designed inserts amplified by PCR with 15-bp extensions complementary to the regions flanking the insertion site (primers are listed in Suppl. Table S2). paGFP-RIM1 and PRM3-B constructs were generated using conventional restriction enzyme-based cloning by digestion and ligation. Chemically competent *E. coli* Stellar cells (Cat. No. 636763, Takara Bio Inc.) were used for transformation with the reaction mixture and were subsequently screened for positive clones. Point mutations in PRM2 and PRM3 were introduced using the QuickChange Lightning Site-Directed Mutagenesis Kit (Agilent Technologies) following the manufacturer’s instructions. All generated plasmids were verified by sequencing (Eurofins Genomics, Germany) and by immunoblotting after transfection into HEK293T cells to ensure that proteins were expressed and had the correct size (Suppl. Fig. S5). Plasmids encoding Myc-RIM-BP2 and EGFP-RIM-BP2 under the CMV promoter were kindly provided by Anna Fejtova (University Erlangen-Nürnberg, Germany; RIM-BP2 sequence described in^77^). Additional constructs used in this study included EGFP-ELKS1αB (kindly provided by Pascal Kaeser, Harvard Medical School, USA; EKS1αB sequence described in^78^), Munc13-1-EGFP (kindly provided by Nils Brose, Max Planck Institute for Multidisciplinary Sciences, Germany; Munc13-1 sequence described in^79^), EGFP-Bassoon (kindly provided by Thomas Dresbach, University Medical Center Göttingen, Germany; EGFP-Bassoon sequence described in^80^), and EGFP-Liprin-α3 (cloned in our laboratory; Liprin-α3 sequence described in^81^).

### Immunocytochemistry of HEK293T cells and primary neurons

Fixed HEK293T cells and neurons transfected with Flag-RIM1 or Myc-RIM-BP2 and seeded on coverslips were permeabilized with 0.3% Triton X-100 (Sigma-Aldrich) in Dulbecco’s phosphate buffered saline (DPBS, Gibco, Thermo Fisher Scientific) for 10 min at RT, and incubated with primary antibodies against RIM1 (1:2000, rabbit, Cat. No. 140013, Synaptic Systems) and Flag-tag (1:1000, mouse, Cat. No. F1804, Sigma-Aldrich), or Myc-tag (1:200, mouse, Cat. No. ab32, Abcam) diluted in 0.1% Triton X-100 overnight at 4 °C. The next day, cells were washed with DPBS and subsequently incubated with the corresponding fluorescent secondary antibodies (1:400; Alexa Fluor 568 goat anti-rabbit (Cat. No. A11011, Invitrogen) and Alexa Fluor 488 goat anti-mouse (Cat. No. A11001, Invitrogen) were used) diluted in 0.1% Triton X-100 for 2 h at RT. Secondary antibodies were then removed by washing, and coverslips were mounted on microscope slides with Mowiol 4-88 and dried overnight at RT.

### Immunoblotting

HEK293T cells transfected with plasmids encoding full-length RIM1 or truncation mutants were washed with DPBS and lysed in lysis buffer (50 mM HEPES, 150 mM NaCl, 1% Triton X-100, pH 7.4) containing a protease inhibitor cocktail (cOmplete, Roche) for 1 h at 4 °C, followed by sonification and centrifugation. Laemmli SDS sample buffer (Thermo Fisher Scientific) was added to the supernatant, and samples were incubated at 95 °C for 5 min. Denatured samples were separated by 8% SDS-polyacrylamide gel electrophoresis and transferred to nitrocellulose membranes. To prevent nonspecific antibody binding, membranes were blocked for 1.5 h in blocking buffer (2% fish gelatin (Sigma-Aldrich) in DPBS). Proteins were detected using the following primary antibodies (2 h incubation at RT) diluted in blocking buffer: anti-GFP antibody (1:1000, rabbit, Cat. No. ab290, Abcam) to detect GFP-fused proteins, and anti-β-actin antibody (1:5000, mouse, Cat. No. ab6276, Abcam) as a loading control. After washing with 0.1% Tween 20 (Thermo Fisher Scientific) in DPBS, blots were incubated for 45 min at RT with IRDye 680RD goat anti-mouse (Cat. No. 926-68070, LICOR-BIO) and IRDye 800CW goat anti-rabbit (Cat. No. 926-32211, LICOR-BIO) antibodies (1:10000), washed again with 0.1% Tween 20, and scanned using an infrared scanning system (Odyssey, LICOR-BIO).

### Photoactivation and imaging of paGFP-RIM1-expressing cells

Live cells in imaging buffer (150 mM NaCl, 4 mM KCl, 2 mM MgCl_2_, 2 mM CaCl_2_, 10 mM D-glucose, 10 mM HEPES) were imaged using a 2 laser/2 scanner two-photon laser-scanning system (Ultima multiphoton platform, Bruker Corporation). Two-photon photoactivation of paGFP-RIM1 (at 750 nm) and acquisition (at 950 nm) were performed using tunable femtosecond Ti: Sapphire Chameleon Vision II lasers (Coherent Corporation) and a Nikon CFI Apo 60× water immersion objective (NA 1.0), with emission collected through a 527/70 nm bandpass filter. Imaging was performed as a continuous time series of frames, while local point photoactivation started after frame 10 (using a parked 750 nm laser beam and a photoactivation time of 5 ms). Time-lapse acquisition at 950 nm excitation was performed at 0.03 s/frame, 100×100 pixel per frame with a pixel size of 0.045 μm (10 pre-activation and 2500 post-activation frames were recorded).

### Confocal imaging

Live-cell imaging was performed in imaging buffer using a laser-scanning inverted confocal microscope (Nikon Eclipse Ti equipped with an A1 confocal controller; Nikon Instruments Inc.) controlled by NIS-Elements software (Nikon Instruments Inc.). Images were acquired using a Nikon Plan Apo IR 60× water-immersion objective (NA 1.27). Excitation was provided by 405, 488, or 568 nm lasers, and fluorescence emission was collected using 450/50 nm, 525/50 nm, or 595/50 nm band-pass filters for Hoechst 33342, GFP/Alexa Fluor 488, and Alexa Fluor 568, respectively. The pinhole diameter was set to 1.2 Airy units (37 μm, referenced to 525/50 nm emission) and kept constant for all acquisitions. The voltage applied to the multi-alkali PMTs and laser power were adjusted individually for each field of view to optimize signal intensity while minimizing the number of saturated pixels. In experiments with varying RIM1 DNA amounts, PMT voltage and laser power were kept constant across all conditions. Images (1024×1024 pixels with a pixel size of 0.207 μm) were acquired using line averaging (2×) and a pixel dwell time of 6.2 µs.

### Quantification of droplet properties and F-ratio

Quantification and analysis of droplet formation were performed using ImageJ software (National Institutes of Health, USA). Regions of interest (ROIs) corresponding to individual transfected cells were manually selected. For each ROI, images were thresholded using the local Phansalkar method in the Auto Local Threshold plugin (radius = 15 pixels, k-value = 0.25, r-value = 0.5). In cases of low droplet-to-nucleoplasm contrast, the threshold was manually adjusted until clear, round droplets were distinguished. Following thresholding, images were converted to binary masks and adjacent droplets were separated using the watershed algorithm implemented in ImageJ. Particle analysis was performed with a minimum size exclusion of 3 pixels (~ 0.13 µm^2^). Area and volume (calculated based on the radius of the equal-area circle) for each detected droplet as well as the droplet number per cell were exported for further analysis.

For F-ratio quantification, 1-pixel-width intensity line profiles were manually drawn in a single-cell ROI across 5 droplets, within the nucleoplasm region devoid of droplets, and outside the transfected cell (background). The mean intensity of the background line profile was subtracted from both the nucleoplasm and droplet profiles. For each droplet, the peak value of its background-corrected intensity profile, which represents a general metric of droplet intensity due to spherical droplet geometry convolved with the point spread function and is related to the concentration of GFP-tagged protein molecules in droplets, was divided by the mean of the background-corrected nucleoplasm profile. The resulting ratios were averaged across droplets to obtain the F-ratio for each analyzed cell. 2D plots describing cell distributions as a function of mean droplet volume and droplet number per cell, generation of the characteristic “FL-RIM1” contour, as well as linear trend fitting between F-ratio or droplet properties and protein sequence length, were performed using Igor Pro 8 (WaveMetrics).

### Statistical analysis

Where applicable, summary statistics are shown as mean ± standard error of the mean (SEM), calculated across independent experiments. Mann-Whitney test and two-way ANOVA with post-hoc Sidak’s multiple comparisons test were applied for statistical analysis, with differences considered significant at *p* < 0.05 (the specific test used is indicated in the figure legends). Statistical analyses were performed using Prism GraphPad (version 6.02, GraphPad Software). The number of independent experiments, the number of acquired images per experiment, and the total number of analyzed cells per construct are indicated in the figure legends and summarized in Suppl. Table S3.

## Supplementary Information

Below is the link to the electronic supplementary material.

Supplementary Material 1

## Data Availability

Further information, resources, and the data supporting the findings of this study can be obtained from Susanne Schoch (susanne.schoch@uni-bonn.de) and Dirk Dietrich (dirk.dietrich@uni-bonn.de) upon reasonable request.
