# Supplementary material for: Dissecting the propensity of RIM1 subdomains to form phase condensates
Source: Sci Rep. 2026 Jun 23;16:19547. doi: 10.1038/s41598-026-56567-6 (PMC13294362; doi:10.1038/s41598-026-56567-6)
Supplement: Supplementary file 1 — Supplementary Material 1 [file 41598_2026_56567_MOESM1_ESM.pdf]

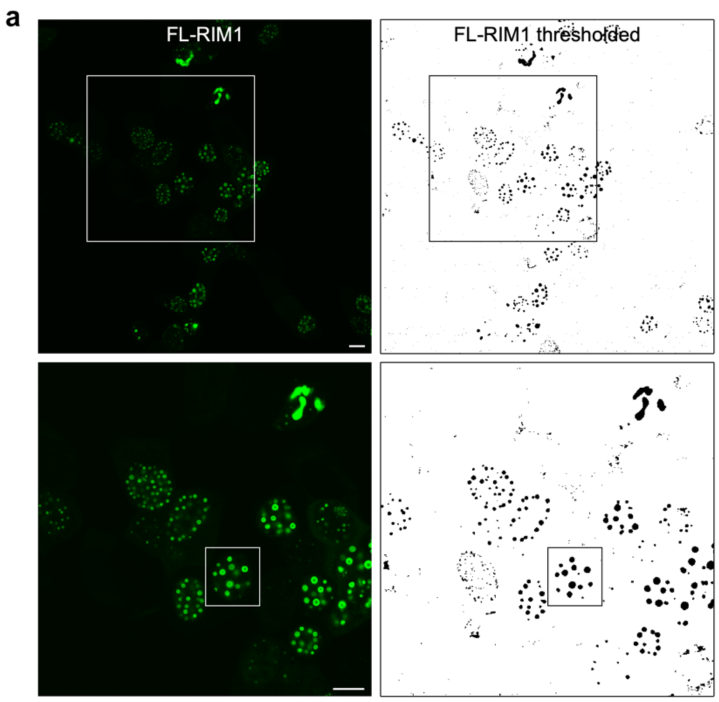

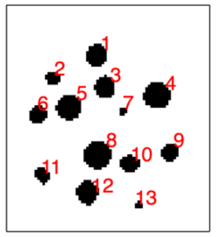

Droplet ID	Droplet area (µm^2)	Droplet volume (µm^3)
1	2.489	2.954
2	1.073	0.836
3	2.36	2.727
4	3.777	5.522
5	3.304	4.518
6	1.674	1.629
7	0.343	0.151
8	4.077	6.193
9	1.674	1.629
10	1.802	1.82
11	1.245	1.045
12	2.704	3.345
13	0.3	0.124

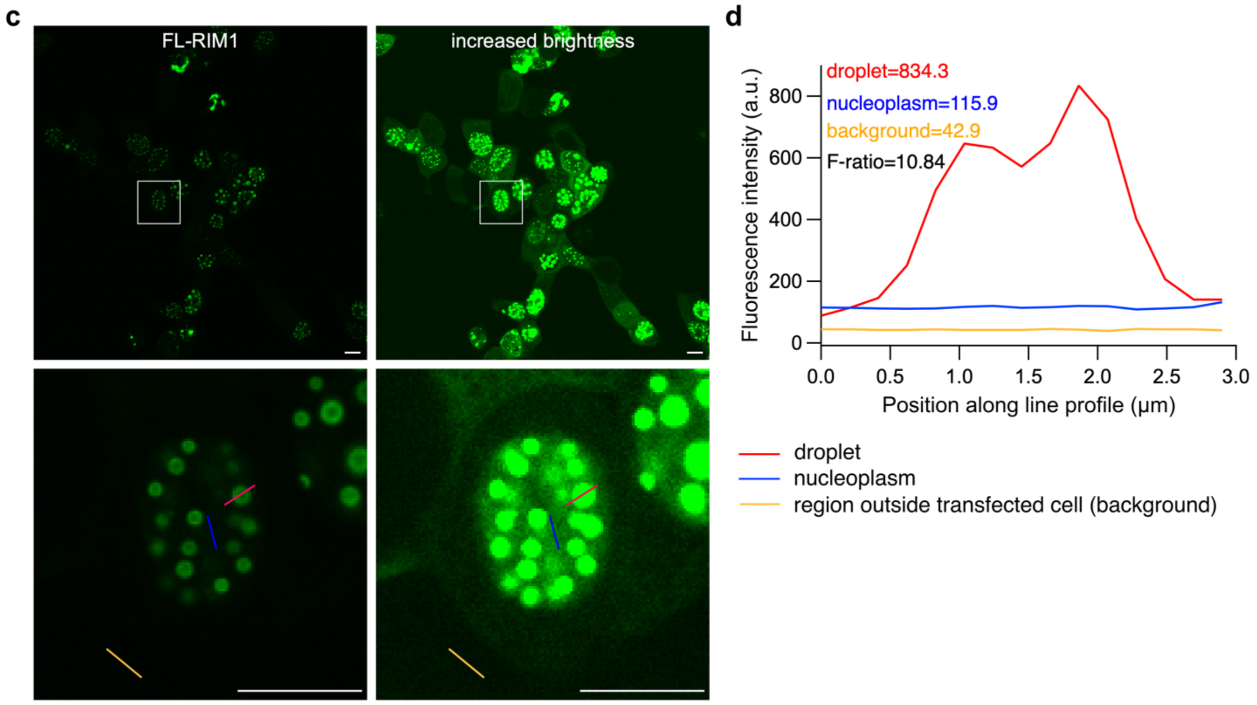

Supplementary Figure S1: Workflow for droplet analysis

(a) Representative confocal images of live HEK293T cells 24 hours after transfection with full-length (FL) RIM1 fused to enhanced GFP (GFP-RIM1), showing characteristic droplets visualized before and after intensity thresholding using a custom ImageJ macro (white pixels are below the set threshold, dark pixels are above). (b) Example of a single HEK293T cell selected from those shown in (a) for quantitative analysis. Droplet number and size were quantified using a custom ImageJ

macro. (c) Representative confocal image of a GFP-RIM1 expressing live HEK293T cell with example line profiles used to measure fluorescent intensities for F-ratio calculation: region outside transfected cells (background, yellow), nucleoplasm (blue), and droplets (red). (d) Graphical representation of the intensity profiles from (c) for F-ratio measurements. The mean background intensity profile (yellow) was subtracted from the nucleoplasm and droplet profiles (blue and red, respectively). The F-ratio was calculated as the peak value of the background-corrected droplet intensity profile divided by the mean of the background-corrected nucleoplasm profile; scale bars = 10 μm.

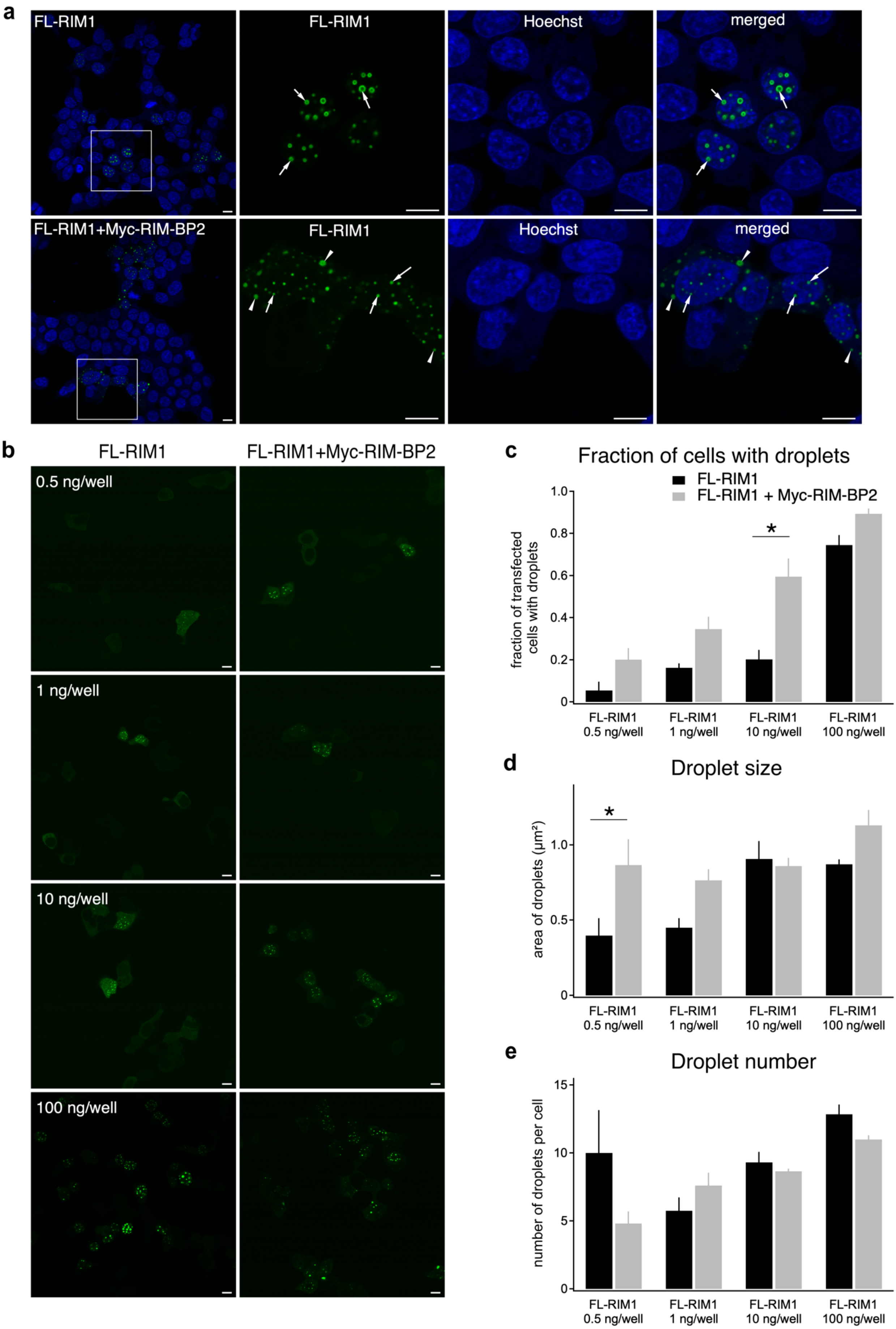
a
FL-RIM1
FL-RIM1
Hoechst
merged
FL-RIM1+Myc-RIM-BP2
FL-RIM1
Hoechst
merged
b
FL-RIM1
FL-RIM1+Myc-RIM-BP2
0.5 ng/well
1 ng/well
10 ng/well
100 ng/well
c
Fraction of cells with droplets
FL-RIM1
FL-RIM1 + Myc-RIM-BP2
fraction of transfected cells with droplets
1.0
0.8
0.6
0.4
0.2
0
*
FL-RIM1 0.5 ng/well
FL-RIM1 1 ng/well
FL-RIM1 10 ng/well
FL-RIM1 100 ng/well
d
Droplet size
area of droplets (µm²)
1.0
0.5
0
*
FL-RIM1 0.5 ng/well
FL-RIM1 1 ng/well
FL-RIM1 10 ng/well
FL-RIM1 100 ng/well
e
Droplet number
number of droplets per cell
15
10
5
0
FL-RIM1 0.5 ng/well
FL-RIM1 1 ng/well
FL-RIM1 10 ng/well
FL-RIM1 100 ng/well

Supplementary Figure S2: Impact of full-length RIM1 DNA amount used for HEK293T cell transfection on the propensity of RIM1 to undergo phase condensation and on RIM-BP's effect on this process

(a) Representative confocal images of PFA-fixed HEK293T cells 24 hours after transfection with GFP-RIM1 alone or co-transfection with GFP-RIM1 and Myc-RIM-BP2, followed by Hoechst staining (100 ng/well of GFP-RIM1 DNA was used, the amount of Myc-RIM-BP2 DNA was adjusted to a 1:1 molar ratio relative to GFP-RIM1). RIM-BP2 promotes droplet formation of RIM1 in the cytoplasm; nuclear condensates are indicated with arrows and cytoplasmic condensates with arrowheads. (b) Representative confocal images of live HEK293T cells 24 hours after transfection with varying amounts of GFP-RIM1 DNA alone or together with Myc-RIM-BP2 (the amount of Myc-RIM-BP2 DNA was adjusted to a 1:1 molar ratio relative to GFP-RIM1), illustrating LLPS behavior across the indicated DNA amounts used for transfection. (c – e) Quantification of the fraction of transfected cells containing droplets (c), droplet area (d), and the number of droplets per cell (e) at different amounts of GFP-RIM1 DNA, either alone or co-transfected with Myc-RIM-BP2; 4 independent experiments and 4 images per replicate, total number of cells with droplets identified per construct (without/with RIM-BP2): RIM1 (0.5 ng): 3/14, RIM1 (1 ng): 9/23, RIM1 (10 ng): 32/81, RIM1 (100 ng): 266/254 (two-way ANOVA identified a significant effect of RIM1 DNA amount on the fraction of transfected cells with droplets, droplet area, and droplet number, while the presence of RIM-BP2 significantly influenced the fraction of cells and droplet size, post-hoc Sidak's multiple comparisons test was performed to assess the effect of RIM-BP2, $^*p < 0.05$); scale bars = 10 μm.

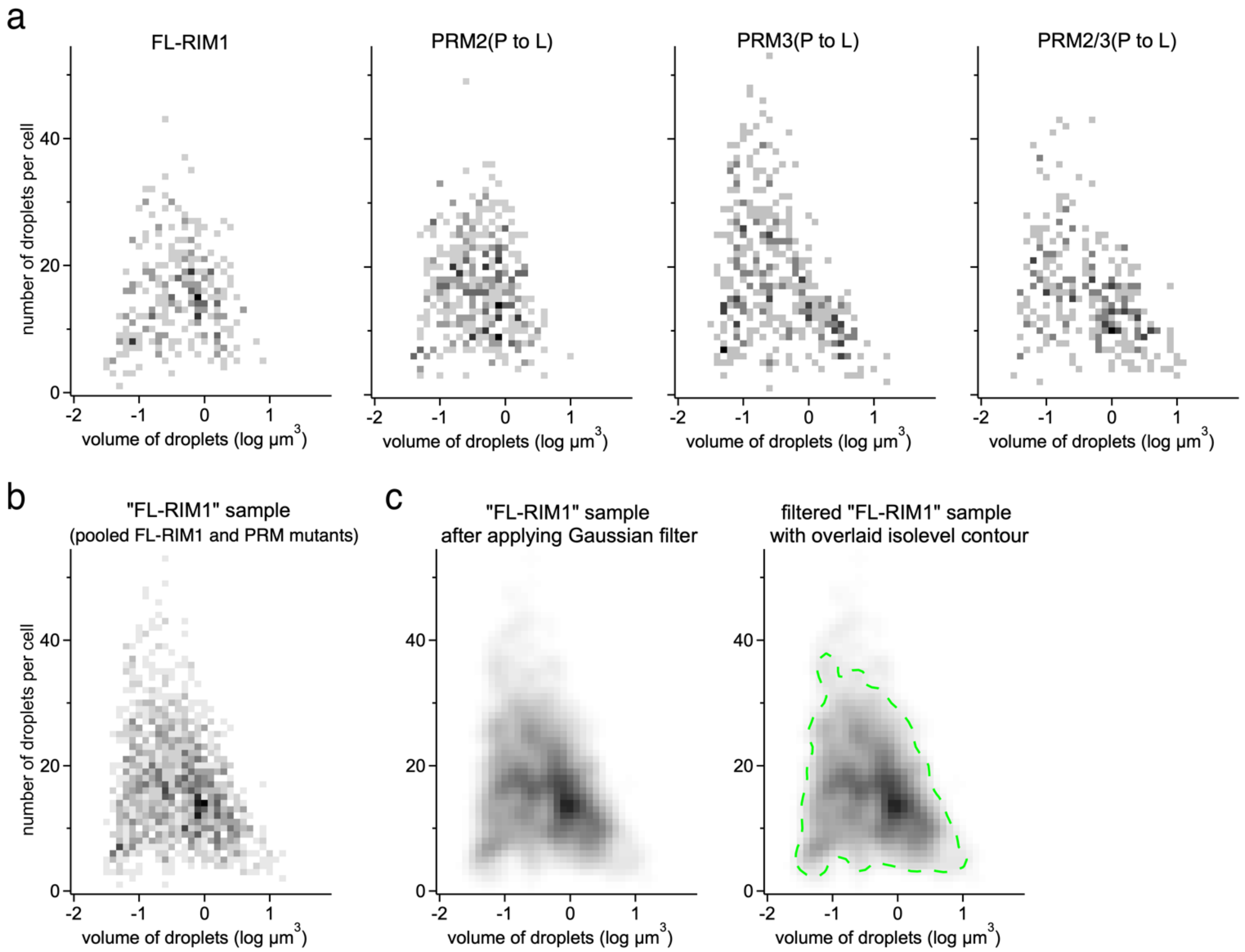

Supplementary Figure S3: Construction of a pooled distribution ("FL-RIM1" sample) and definition of isolevel contour lines of RIM1 droplet properties from FL-RIM1 and PRM mutant datasets

(a) Two-dimensional histograms showing the distribution of cells as a function of mean droplet volume (log_{10}-transformed values) and droplet number per cell for FL-RIM1 and its proline-to-leucine mutants (bin width: 0.1 for log droplet volume and 1 for droplet number per cell; pixel intensity is depicted relative to the number of cells containing droplets of a given mean volume and number, ranging from 0 (white) to the maximum number of cells observed (black)). (b) Two-dimensional histogram of the robust "FL-RIM1" sample, constructed by pooling datasets from FL-RIM1 and all PRM mutants. (c) The "FL-RIM1" histogram was smoothed using a Gaussian filter (kernel size = 5×5 bins, corresponding to $\sigma = 0.1227$ for the log volume axis and $\sigma = 1.227$ for the droplet number axis) to reduce sampling noise. An isolevel contour line was defined at 10% of the maximum bin count and superimposed to all 2D histograms throughout the manuscript for comparison.

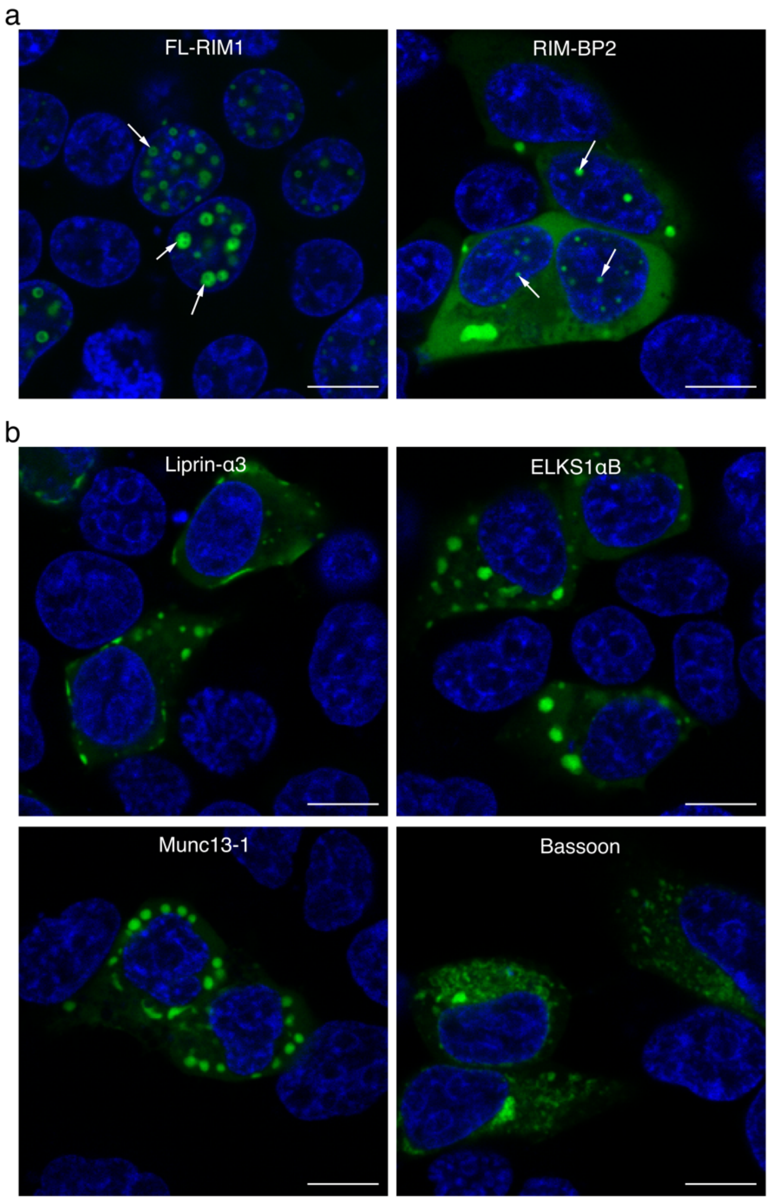

Supplementary Figure S4: Full-length RIM1 and RIM-BP2, but not other tested AZ proteins, form nuclear condensates in HEK293T cells

(a) Representative single-plane confocal images of live Hoechst-stained HEK293T cells expressing FL-RIM1 or RIM-BP2, both of which form nuclear condensates (marked by arrows). (b) Representative single-plane confocal images of live Hoechst-stained HEK293T cells expressing Liprin-α3, ELKS1αB, Munc13-1, or Bassoon. These AZ proteins are largely restricted to the cytoplasm and do not form nuclear condensates; all proteins are fused to enhanced GFP, images show merged Hoechst (blue) and GFP (green) signals; scale bars = 10 μm.

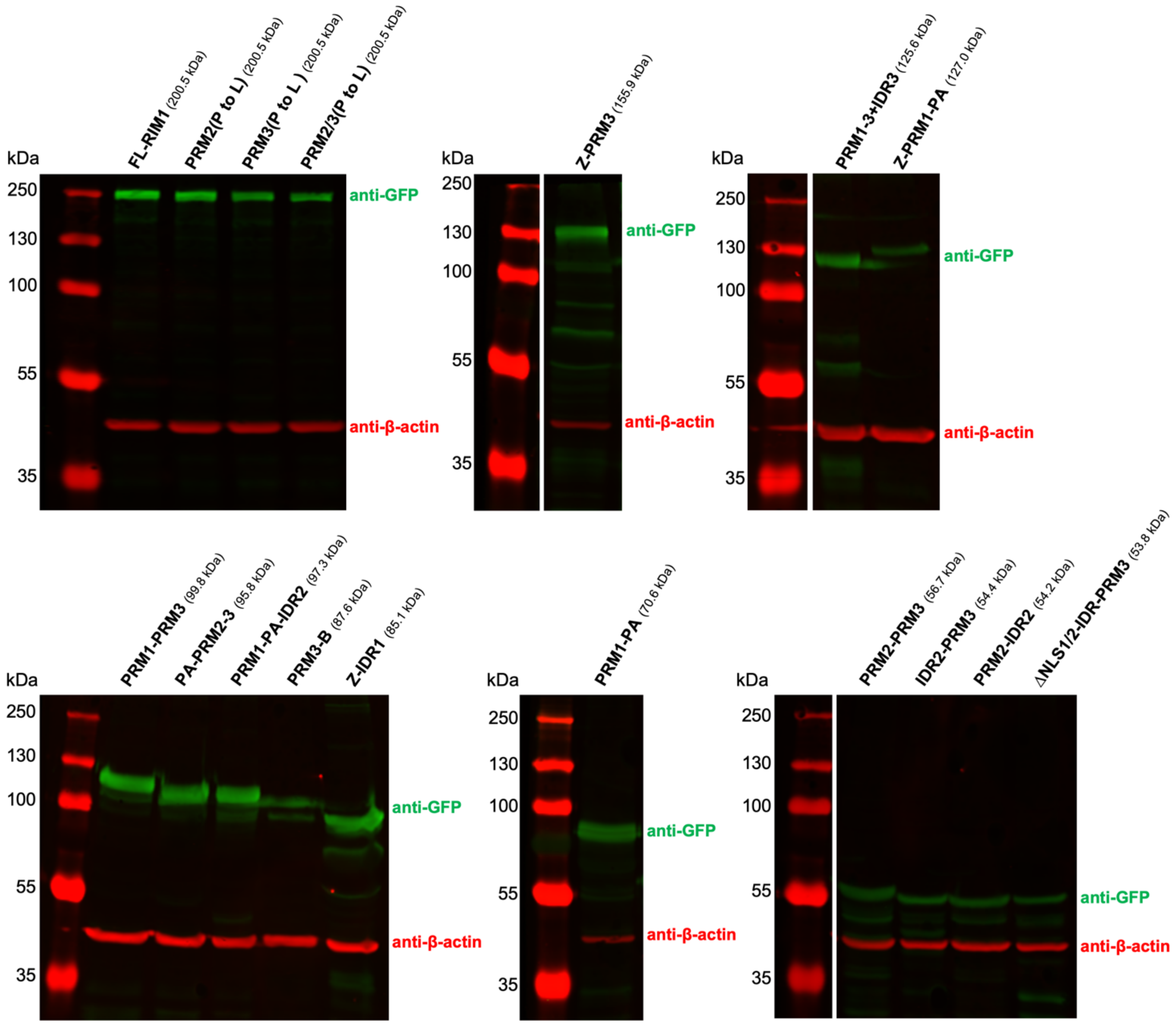

Supplementary Figure S5: Verification of RIM1 mutant expression in HEK293T cells by Western blot

Representative immunoblots of GFP-tagged FL-RIM1 and its mutants expressed in HEK293T cells (cells were harvested 48 hours after transfection). RIM1-derived constructs were detected using an anti-GFP antibody (shown in green), and β-actin was detected as a loading control using an anti-β-actin antibody (shown in red). Images from the two detection channels were merged. The expected molecular weights of each protein, including GFP and linker sequences, are indicated. Molecular weight markers and sample lanes shown within each panel originate from the same blot (cropping of noncontiguous regions of the same blot is indicated by white space). The corresponding full-length blots used to assemble these panels are shown in Supplementary Figure S6.

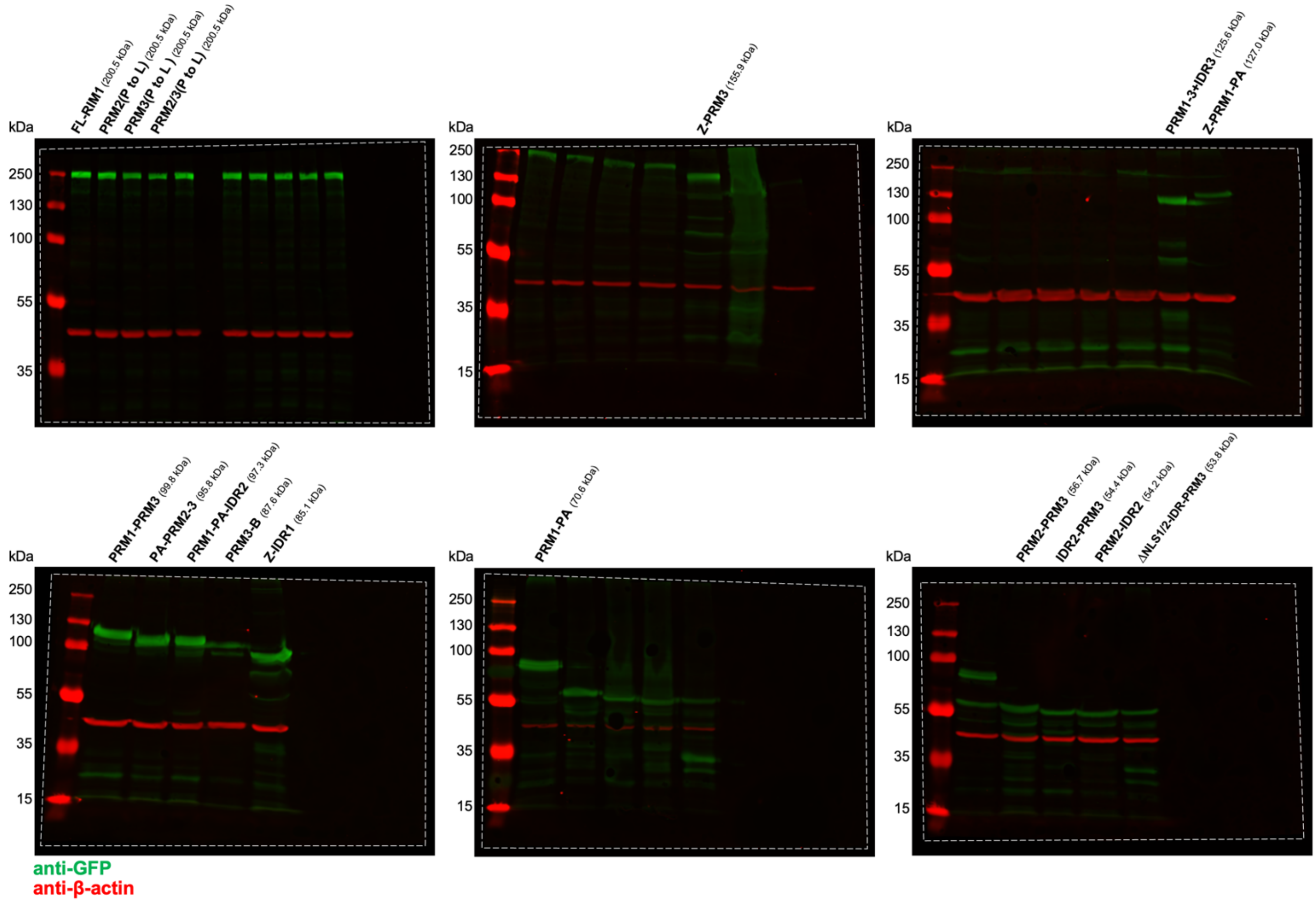

Supplementary Figure S6: Original full-length blots used to assemble Supplementary Figure S5

Original uncropped blots used to assemble the immunoblot panels shown in Supplementary Figure S5 (GFP-tagged FL-RIM1 and its mutants expressed in HEK293T cells were detected using an anti-GFP antibody, and β-actin was detected as a loading control using an anti-β-actin antibody; membrane edges are delineated by white dashed lines; lanes corresponding to those shown in Supplementary Figure S5 are labeled identically).

Supplementary Data S1: Protein sequence of full-length RIM1 with annotated domains and sequence features

Domains: **Zinc finger**, **PDZ**, **C2A**, **C2B** (based on UniProt annotation of rat RIM1, accession number: F1LYS1);

IDRs: **IDR1**, **IDR2**, **IDR3** (predicted using IUPred3 [1]);

PRMs: **PRM1**, **PRM2**, **PRM3** (defined as SH3-domain binding motifs predicted using MoDPepInt [2]);

NLSs: **NLS1**, **NLS2** (predicted using cNLS Mapper [3] and NLSdb [4]);

SH3-binding consensus motifs (where prolines (**P**) are mutated to leucine in PRM mutants) are boxed**.**

1 MSSAVGPRGPRPPTVPPPMQELPDLSHLTEEERNIIMAVMDRQKEEEEKEEAMLKCVVRDMAKPAACK 68

69 TPRNAESQPHQPPLNIFRCVCVPRKPSSEEGGPERDWRLHQQFESYKEQVRKIGEEARRYQGEHKDDAP 137

138 TCGICHKTKFADGCGHLCSYCRTKFCARCGGRVSLRSNNEDKVVMWVCNLCRKQQEILTKSGAWFFGSG 206

207 PQQPSQDGTLSDTATGAGSEVPREKKARLQERSRSQTPLSTAAVSSQDTATPGAPLDRNKGAEPSQQALG 276

277 PEQKQASRSRSEPPRERKKAPGLSEQNGKGGQKSERKRVPKSVVQPGEGIADERERKERRETRRLEKGRS 346

347 QDYSDRPEKRDNGRVAEDQKQRKEEEYQTRYRSDPNLARYPVKAPPEEQQMRMHARVSRARHERRHS 413

414 DVALPHTEAAAAAPAEATAGKRAPATARVSPPESPRARAPAAQPPTEHGPPPPRPAPGPAEPPEPRVPEPL 484

485 RKQGRLDPGSAVLLRKAKREKAESMLRNDSLSSDQSESVRPSPPKPHRPKRGGKRRQMSVSSSEEEGVST 554

555 PEYTSCEDVELESESVSEKGDLDYYWLDPATWHSRETSPISSHPVTWQPSKEGDRLIGRVILNKRTTMPKE 625

626 SGALLGLKVVGGKMTDLGRLGAFITKVKKGSLADVVGHLRAGDEVLEWNGKPLPGATNEEVYNIILESKSE 696

697 PQVEIIVSRPIGDIPRIPRSSHPPLESSSSSFESQKMERPSISVISPTSPGALKDAPQVLPGQLSVKLWYDKVG 770

771 HQLIVNVLQATDLPPRVDGRPRNPYVKMYFLPDRSDKSKRRTKTVKKLLEPKWNQTFVYSHVHRRDFRE 839

840 RMLEITVWDQPRVQDEESEFLGEILIELETALLDDEPHWYKLQTHDESSLPLPQPSPFMPRRHIHGESSSK 910

911 KLQRSQRISDSDISDYEVDDGIGVVPPVGYRASARESKATTLTVPEQQRTTHHRSRSVSPHRGDDQGRPR 980

981 SRLPNVPLQRSLDEIHPTRRSRSPTRHHDASRSPVYHRSRHVESQYSSEPDSELLMLPRAKRGRSAESLHM 1051

1052 TSELQPSLDRARSASTNCLRPDTSLHSPERERHSRKSERCSIQKQSRKGTASDADRTHRQGSPTQSPPADTS 1123

1124 FGSRRGRQLPQVPVRSGSIEQASLVVEERTRQMKMKVHRFKQTTGSGSSQELDHEQYSKYNIHKDQYRS 1192

1193 CDNASAKSSDSDVSDVSAISRASSTSRLSSTSFMSEQSERPRGRISSFTPKMQGRRMGTSGRAIIKSTSVSG 1264

1265 EIYTLERNDGSQSDTAVGTVGAGGKKRRSSLSAKVVAIVSRRSRSTSQLSQTESGHKKLKSTIQRSTETGMA 1336

1337 AEMRKMVRQPSRESTDGSINSYSSEGNLIFPGVRVGPDSQFSDFLDGLGPAQLVGRQTLATPAMGDIQIG 1406

1407 MEDKKGQLEVEVIRARSLTQKPGSKSTPAPYVKVYLLENGACIAKKKTRIARKTLDPLYQQSLVFDESPQGK 1478

1479 VLQVIVWGDYGRMDHKCFMGVAQILLEELDLSSMVIGWYKLFPPSSLVDPTLAPLTRRASQSSLESSSGPP 1549

1550 CIRS 1553

Supplementary Table S1: Amino acid positions (start-end) of RIM1 truncation mutants numbered relative to the full-length RIM1 sequence

RIM1 mutant	Amino acid positions
PRM (P to L) mutants	1-1553
Z-PRM3	1-1144
Z-PRM1-PA	1-888
Z-IDR1	1-517
PRM1-3+IDR3	504-1380
PRM1-PRM3	504-1144
PRM1-PA-IDR2	504-1121
PRM1-PA	504-885
PA-PRM2-3	540-1144
PRM2-PRM3	885-1144
PRM2-IDR2	885-1121
IDR2-PRM3	905-1144
PRM3-B	1015-1553
ΔNLS1/2-IDR-PRM3	1047-1286
ΔNLS1-PRM3-B	1108-1553
ΔNLS1/2-B	1317-1553

Supplementary Table S2: Primers used for cloning RIM1-derived constructs

Construct	Primer sequence
EGFP-Z-PRM3	*fw:*GCGGAATTCTATGTCCTCGGCCGTGGG
	*rev:*GCGCATATGTCATTGTTCTATACTGCCGCTTC
EGFP-PRM1-3+IDR3	*fw:*GCGGAATTCGGAGAAGGCGGAGAGCATG
	*rev:*GCGCATATGCTAGAAATCACTGAACTGACTGTC
EGFP-PRM1-PRM3	*fw:*GCGGAATTCGGAGAAGGCGGAGAGCATG
	*rev:*GCGCATATGTCATTGTTCTATACTGCCGCTTC
EGFP-PA-PRM2-3	*fw:*GCGGAATTCGCGTCAGATGTCGGTGAGCAG
	*rev:*GCGCATATGTCATTGTTCTATACTGCCGCTTC
EGFP-PRM1-PA-IDR2	*fw:*GCGGAATTCGGAGAAGGCGGAGAGCATG
	*rev:*GCGCATATGTCAGTCTGCTGGAGGAGACTGGG
EGFP-PRM1-PA	*fw:*GCGGAATTCGGAGAAGGCGGAGAGCATG
	*rev:*GCGCATATGTTAGTCATGTGTCTGGAGTTTATACC
EGFP-PRM2-PRM3	*fw:*GCGGAATTCGGACGAATCTTCACTACCTCTG
	*rev:*GCGCATATGTCATTGTTCTATACTGCCGCTTC
EGFP-IDR2-PRM3	*fw:*GCGGAATTCGGGAGAGAGCTCCAGCAAAAAG
	*rev:*GCGCATATGTCATTGTTCTATACTGCCGCTTC
EGFP-PRM2-IDR2	*fw:*GCGGAATTCGGACGAATCTTCACTACCTCTG
	*rev:*GCGCATATGTCAGTCTGCTGGAGGAGACTGGG
EGFP-Z-PRM1-PA	*fw:*CGGGGCCGGGGAATTGGATGTCCTCGGCC
	*rev:*CAGGTCCCGGGCATATCAGTCATGTGTCTGGAGTTTATACCAATGG
EGFP-Z-IDR1	*fw:*CGGGGCCGGGGAATTGGATGTCCTCGGCC
	*rev:*CAGGTCCCGGGCATATCACTCGGACTGATCGGAGC
EGFP-ΔNLS1-PRM3-B	*fw:*CCTAGGTACGTAGTATACACGCACCGACAAGGAAGC
	*rev:*GGATCCCCGGGCATATGTCAT
EGFP-ΔNLS1/2-B	*fw:*CCTAGGTACGTAGTATACGAGTCGGGCCACAAGAAGTT
	*rev:*GGATCCCCGGGCATATGTCAT
EGFP-ΔNLS1/2-IDR-PRM3	*fw:*GGGGCCGGGGAATTCAGAAAGCCTACACATGACCAGTGAAC
	*rev:*CAGGTCCCGGGCATATCAGGCTCCGACGGTACCT
EGFP-PRM2 (P to L)	*fw:*CTGCCTCAGCCATCACTGTTCATGCTCAGGCGGCATATTCATGGAGAG
	*rev:*CTCTCCATGAATATGCCGCCTGAGCATGAACAGTGATGGCTGAGGCAG
EGFP-PRM3 (P to L)	*fw:*CGGCAGTCGCGTGGAAGACAGCTCCTACAGGTGCTAGTTCGAAGCGGCAG
	*rev:*CTGCCGCTTCGAACTAGCACCTGTAGGAGCTGTCTTCCACGGCGACTGCC

Supplementary Table S3: Summary of quantitative droplet measurements

Construct	Parameter (unit)	Mean (between experiments)	SEM (between experiments)	Number of experiments; total number of analysed cells	Figure reference
Cells transfected with FL-RIM1 and its mutants					
FL-RIM1 (GFP-tag)	number of droplets per cell	15.52	0.64	3; 272	Fig.1d
	droplet area (μm^2)	0.97	0.08		Fig.1e
FL-RIM1 (Flag-tag)	number of droplets per cell	14.85	0.47	3; 519	Fig.1d
	droplet area (μm^2)	1.35	0.06		Fig.1e
FL-RIM1 (GFP-tag)	number of droplets per cell	3.01	0.54	3; 49	Fig.3b
	droplet area (μm^2)	2.61	0.4		Fig.3c
FL-RIM1 (Flag-tag)	number of droplets per cell	2.08	0.23	3; 104	Fig.3b
	droplet area (μm^2)	2.88	0.31		Fig.3c
FL-RIM1	number of droplets per cell	15.52	0.64	3; 272	Fig.4b + Fig.9b
	log(droplet volume (μm^3))	-0.327	0.055		Fig.4b + Fig.9c
	log(F-ratio)	1.218	0.064	3; 60	Fig.9a
PRM2 (P to L)	number of droplets per cell	16.60	0.93	3; 388	Fig.4b + Fig.9b
	log(droplet volume (μm^3))	-0.344	0.038		Fig.4b + Fig.9c
	log(F-ratio)	1.215	0.080	3; 60	Fig.9a
PRM3 (P to L)	number of droplets per cell	17.93	2.27	3; 370	Fig.4b + Fig.9b
	log(droplet volume (μm^3))	-0.407	0.139		Fig.4b + Fig.9c
	log(F-ratio)	1.297	0.096	3; 60	Fig.9a
PRM2/3 (P to L)	number of droplets per cell	15.68	0.72	3; 292	Fig.4b + Fig.9b
	log(droplet volume (μm^3))	-0.286	0.078		Fig.4b + Fig.9c
	log(F-ratio)	1.163	0.057	3; 60	Fig.9a
Z-PRM3	number of droplets per cell	10.46	1.09	3; 253	Fig.5b + Fig.9b
	log(droplet volume (μm^3))	-0.500	0.049		Fig.5b + Fig.9c
	log(F-ratio)	0.913	0.016	3; 60	Fig.9a
PRM1-3+IDR3	number of droplets per cell	12.28	1.05	3; 129	Fig.5b + Fig.9b
	log(droplet volume (μm^3))	-0.625	0.065		Fig.5b + Fig.9c
	log(F-ratio)	0.832	0.091	3; 60	Fig.9a
PRM1-PRM3	number of droplets per cell	8.23	0.49	3; 243	Fig.5b + Fig.9b
	log(droplet volume (μm^3))	-0.009	0.091		Fig.5b + Fig.9c
	log(F-ratio)	0.432	0.028	3; 60	Fig.9a
Z-PRM1-PA	number of droplets per cell	15.57	1.59	3; 337	Fig.5b + Fig.9b
	log(droplet volume (μm^3))	-0.257	0.077		Fig.5b + Fig.9c
	log(F-ratio)	0.696	0.021	3; 60	Fig.9a
Z-IDR1	number of droplets per cell	11.74	0.72	3; 120	Fig.5b + Fig.9b
	log(droplet volume (μm^3))	-0.144	0.143		Fig.5b + Fig.9c
	log(F-ratio)	0.165	0.016	3; 60	Fig.9a
PRM3-B	number of droplets per cell	17.88	0.88	3; 146	Fig.5b + Fig.9b
	log(droplet volume (μm^3))	-0.015	0.028		Fig.5b + Fig.9c
	log(F-ratio)	0.441	0.009	3; 60	Fig.9a

PA-PRM2-3	number of droplets per cell	8.88	0.74	3; 189	Fig.6b + Fig.9b
	log(droplet volume (μm^3))	0.130	0.079		Fig.6b + Fig.9c
	log(F-ratio)	0.487	0.047	3; 60	Fig.9a
PRM1-PA-IDR2	number of droplets per cell	7.31	0.84	3; 87	Fig.6b + Fig.9b
	log(droplet volume (μm^3))	0.076	0.020		Fig.6b + Fig.9c
	log(F-ratio)	0.280	0.016	3; 60	Fig.9a
PRM2-PRM3	number of droplets per cell	12.50	1.56	3; 198	Fig.6b + Fig.9b
	log(droplet volume (μm^3))	-0.192	0.063		Fig.6b + Fig.9c
	log(F-ratio)	0.314	0.015	3; 60	Fig.9a
IDR2-PRM3	number of droplets per cell	8.19	0.85	3; 117	Fig.6b + Fig.9b
	log(droplet volume (μm^3))	-0.294	0.038		Fig.6b + Fig.9c
	log(F-ratio)	0.200	0.027	3; 60	Fig.9a
PRM2-IDR2	no droplets observed				Fig.6c
PRM1-PA	no droplets observed				Fig.6c
*Cells transfected with FL-RIM1 and its mutants without (-) or with (**+**) Myc-RIM-BP2 co-transfection*					
FL-RIM1 (-)	number of droplets per cell	15.35	0.35	3; 374	Fig.4d
	log(droplet volume (μm^3))	-0.338	0.055		
FL-RIM1(+)	number of droplets per cell	11.26	0.38	3; 405	Fig.4d
	log(droplet volume (μm^3))	-0.331	0.023		
PRM2-PRM3 (-)	number of droplets per cell	13.40	0.81	3; 198	Fig.7b
	log(droplet volume (μm^3))	-0.232	0.045		
PRM2-PRM3 (+)	number of droplets per cell	7.94	0.79	3; 392	Fig.7b
	log(droplet volume (μm^3))	0.129	0.076		
IDR2-PRM3 (-)	number of droplets per cell	8.14	0.69	3; 117	Fig.7c
	log(droplet volume (μm^3))	-0.289	0.060		
IDR2-PRM3 (+)	number of droplets per cell	7.12	1.34	3; 315	Fig.7c
	log(droplet volume (μm^3))	0.118	0.079		
PRM2-IDR2 (-)	no droplets observed				Fig.7d
PRM2-IDR2 (+)	number of droplets per cell	5.17	1.14	3; 59	Fig.7d
	log(droplet volume (μm^3))	0.041	0.037		
PRM1-PA-IDR2 (-)	number of droplets per cell	6.45	0.11	3; 87	Fig.7e
	log(droplet volume (μm^3))	-0.073	0.081		
PRM1-PA-IDR2 (+)	no droplets observed				Fig.7e
FL-RIM1, 0.5 ng/well; (-)	fraction of cells with droplets	0.054	0.041	4; 3	Fig.S2c
	droplet area (μm^2)	0.40	0.11		Fig.S2d
	number of droplets per cell	10	3.14		Fig.S2e
FL-RIM1, 0.5 ng/well; (+)	fraction of cells with droplets	0.201	0.054	4; 14	Fig.S2c
	droplet area (μm^2)	0.87	0.17		Fig.S2d
	number of droplets per cell	4.81	0.89		Fig.S2e
FL-RIM1, 1 ng/well; (-)	fraction of cells with droplets	0.162	0.020	4; 9	Fig.S2c
	droplet area (μm^2)	0.45	0.06		Fig.S2d
	number of droplets per cell	5.75	0.97		Fig.S2e

FL-RIM1, 1 ng/well; (+)	fraction of cells with droplets	0.346	0.058	4; 23	Fig.S2c
	droplet area (μm^2)	0.76	0.07		Fig.S2d
	number of droplets per cell	7.59	0.94		Fig.S2e
FL-RIM1, 10 ng/well; (-)	fraction of cells with droplets	0.202	0.044	4; 32	Fig.S2c
	droplet area (μm^2)	0.91	0.12		Fig.S2d
	number of droplets per cell	9.31	0.77		Fig.S2e
FL-RIM1, 10 ng/well (+)	fraction of cells with droplets	0.595	0.086	4; 81	Fig.S2c
	droplet area (μm^2)	0.86	0.05		Fig.S2d
	number of droplets per cell	8.65	0.18		Fig.S2e
FL-RIM1, 100 ng/well; (-)	fraction of cells with droplets	0.744	0.047	4; 266	Fig.S2c
	droplet area (μm^2)	0.87	0.03		Fig.S2d
	number of droplets per cell	12.84	0.71		Fig.S2e
FL-RIM1, 100 ng/well; (+)	fraction of cells with droplets	0.893	0.024	4; 254	Fig.S2c
	droplet area (μm^2)	1.13	0.10		Fig.S2d
	number of droplets per cell	10.99	0.30		Fig.S2e

References

1. Erdős, G., Pajkos, M. & Dosztányi, Z. IUPred3: Prediction of protein disorder enhanced with unambiguous experimental annotation and visualization of evolutionary conservation. *Nucleic Acids Res.* **49,** W297–W303 (2021).

2. Kundu, K., Mann, M., Costa, F. & Backofen, R. MoDPepInt: An interactive web server for prediction of modular domain-peptide interactions. *Bioinformatics* **30,** 2668–2669 (2014).

3. Kosugi, S., Hasebe, M., Tomita, M. & Yanagawa, H. Systematic identification of cell cycle-dependent yeast nucleocytoplasmic shuttling proteins by prediction of composite motifs. *Proc. Natl. Acad. Sci.* **106,** 10171–10176 (2009).

4. Nair, R., Carter, P. & Rost, B. NLSdb: Database of nuclear localization signals. *Nucleic Acids Res.* **31,** 397–399 (2003).
